# Detecting bots in social-networks using node and structural embeddings

**DOI:** 10.1186/s40537-023-00796-3

**Published:** 2023-07-19

**Authors:** Ashkan Dehghan, Kinga Siuta, Agata Skorupka, Akshat Dubey, Andrei Betlen, David Miller, Wei Xu, Bogumił Kamiński, Paweł Prałat

**Affiliations:** 1 Toronto Metropolitan University, Toronto, ON Canada; 2 SGH Warsaw School of Economics, Warsaw, Poland; 3 Patagona Technologies, Pickering, ON Canada

**Keywords:** Detecting bots, Node and structural embeddings, Social networks

## Abstract

Users on social networks such as Twitter interact with each other without much knowledge of the real-identity behind the accounts they interact with. This anonymity has created a perfect environment for bot accounts to influence the network by mimicking real-user behaviour. Although not all bot accounts have malicious intent, identifying bot accounts in general is an important and difficult task. In the literature there are three distinct types of feature sets one could use for building machine learning models for classifying bot accounts. These feature-sets are: user profile metadata, natural language features (**NLP**) extracted from user tweets and finally features extracted from the the underlying social network. Profile metadata and **NLP** features are typically explored in detail in the bot-detection literature. At the same time less attention has been given to the predictive power of features that can be extracted from the underlying network structure. To fill this gap we explore and compare two classes of embedding algorithms that can be used to take advantage of information that network structure provides. The first class are classical embedding techniques, which focus on learning proximity information. The second class are structural embedding algorithms, which capture the local structure of node neighbourhood. We show that features created using structural embeddings have higher predictive power when it comes to bot detection. This supports the hypothesis that the local social network formed around bot accounts on Twitter contains valuable information that can be used to identify bot accounts.

## Introduction

Internet and social media impact all aspects of our lives. We use them to read news, connect with friends and family, share opinions, buy products, and entertain us. It affects our beliefs, behaviour and so it shapes our political, financial, health, and other important decisions. Unfortunately, as a result, social networks created an information platform in which automated accounts (including human-assisted bot accounts and bot-assisted humans) can try to take advantage of the system for various opportunistic reasons: trigger collective attention [12, 28], gain status [10, 43], monetize public attention [9], diffuse disinformation [5, 16, 34], or seed discord [47]. It is known that a large fraction of active Twitter users are bots [44] and they are responsible for much disinformation—see, [48] for many examples of manipulation of public opinion. Having said that, not all bot accounts are designed to harm or take advantage of other users. Some of them are legitimate and useful tools such as chatbots that respond to common questions of users, or knowbots that are designed to automatically retrieve some useful information from the Internet. On the other hand, human accounts may also spread disinformation and be responsible for some other malicious behaviour. Detecting bots and understanding roles they play within the system falls into a common machine learning task of node classification. It is important to note that in this work, we do not focus on the intent of the bot accounts (whether they are benign or malicious); determining this is outside the scope of our study.

The objectives of this paper are to investigate: (1) whether graph embeddings extract information from the associated network that can be successfully used for node classification task, (2) what is the relative value of classical vs. structural node embeddings for bot detection, (3) does the predictive power of embeddings depend on their complexity (measured by the dimension of the embedding). To achieve these goals, we start with defining classical and structural embedding techniques. Classical embedding techniques, such as Node2Vec [21] and DeepWalk [37] learn information related to proximity of nodes in the network. On the other hand, structural embedding algorithms such as Role2Vec [1] and Struc2Vec [39] learn representations of the local structure surrounding each node. In our work, we build features using both classical and structural embedding techniques and use those features to train models for classifying bots.

In our experiments, we concentrate on Twitter data and the task of identifying bot accounts, but our questions (and answers) are broader and so potentially more influential. They are applicable to all kinds of networks and data sets that are naturally represented as graphs which, of course, includes social media platforms such as Twitter. Moreover, they are applicable to a much wider class of machine learning tasks: node classification algorithms train a model to learn in which class a node of the graph belongs to. Bot detection is a specific example of this class of problems in which a binary classification is performed (nodes are categorized into bots and humans). However, in general, multi-class classifications is also often considered and needed. Other important applications of this nature include, for example, identifying nodes associated with users that might be interested in some specific product, or detecting hostile actors. For this reason there is an increasing need for effective methods of analysis data represented as graphs. For more details we direct the reader to a recent survey [30] and a book [25]. Lastly, we point out that although our study focuses on bot detection on Twitter social network, bot detection in general is a domain specific task. Users (including bots) on other social networks may interact with one another in different ways, which could impact the predictive information captured by the underlying social network.

There are many approaches that can be used to perform node classification in graphs. Most techniques attempt to detect bots at the account level by processing many social media posts and analyzing them using various NLP techniques with the goal to extract some important and distinguishing features. These features are usually complemented with user metadata, friend metadata, content and language sentiment, as well as temporal features [13]. In this paper, we will refer to these features as **NLP** and **P** (Profile). These techniques are very powerful, but a supervised machine learning algorithm is only as good as the data used for training. Unfortunately, good quality datasets with the ground-truth are rarely available. Additional challenge is that bot accounts evolve rapidly and so one needs to constantly update datasets and evolve the set of features to keep up with the other side. In particular, hot topics discussed on social media evolve rapidly; for example, **NLP** features that were important for bot detection before presidential elections in some country might become quickly outdated after the election. Similarly, results of NLP analysis cannot be easily transferred from one language to another or across differ geographical regions or countries. Furthermore, recent developments in Large-Language-Models (LLMs) such as GPT-4 [35] will make it more difficult to detect human versus bot generated language. Language derived features, which may have helped identify bots, may be obsolete as bots take advantage of more sophisticated LLMs. As a result, a collaborated effort of many researchers and data scientists is needed to maintain bot detection models. One successful example is *Botometer*, bot detection tool developed at Indiana University using various labelled datasets and 1, 209 features (current, 3rd version of the model) [48]; see also [42] for a new supervised learning method that trains classifiers specialized for each class of bots and combines their decisions through the maximum rule (ensemble approach). *Botometer* handles over a quarter million requests every day! However, since the bot score is intended to be used with English-language accounts, what can one do with non-English accounts? What if the content or metadata is not easily available? Finally, how about other node classification tasks which cannot enjoy such powerful tools such as *Botometer*?

An alternative approach is to use some features of nodes that can be calculated exclusively using graph data. The main advantage of this approach is that such information is easier to obtain and is typically less sensitive as it does not include the analysis of user messages and metadata associated with them. More importantly, it can be hypothesised that the signal is more stable in time and graph space, that is, if some topological structure of the network indicates that some nodes are likely to be bots, then such signal is likely to loose its predictive power slower than, for example, discussion topics extracted from **NLP** features. Typical features concentrate on local properties of nodes such as node degree, various node centralities, local clustering coefficient, etc. We will call features derived using this approach as **GF** (**G**raph **F**eatures). The idea behind is that bots need to use some strategies to form an audience. They employ various algorithms to gather followers and expand their own social circles such as following popular accounts and ask to be followed back [2], generating specific content around a given topic with the hope to gain trust and catch attention [17], or even interacting with other users by engaging in conversation [24]. These algorithms create networks around the bots that should be structurally and topologically distinguishable from the ones around real human beings which, in turn, affect the extracted graph features. The same rationale applies to other applications of node classification.

The above approach, based on analysis of predefined graph features, was proved to be useful in various node classifications tasks but it has a few issues. First of all, very often features of one node alone are not enough to adequately classify the node. Indeed, bots typically work in a coordinated way and are not usually suspicious when considered individually. Hence, bot detection requires combining information about multiple bots and analyzing them together [11]. This often is very challenging, both conceptually as well as computationally, as it requires to consider at least a quadratic number of pairs of nodes. features capture properties that are rather local whereas some embedding algorithms aim to extract more global and structural properties. properties Moreover, we often do not have access to a complete network but rather sample it using some sampling method. Unfortunately, the choice of a sampling algorithm may substantially affect **GF**. The features that are to be analyzed need to be predefined by the analyst. Therefore, the result of this approach depends heavily on skills, knowledge, or just sheer luck of the user.

To solve at least some of these problems, we propose to utilize node embedding algorithms that assign nodes of a graph to points in a low dimensional space of real numbers. The goal of the embedding is to decrease the dimension but, at the same time, to extract the most important features of nodes and ignore noise. We will call features obtained based on this approach **EMB**. As mentioned, we consider two classes of embedding techniques. One, which we call classical embeddings, focus on learning local and global proximity information about nodes. Such techniques can be used to identify communities and groups in networks. The second class of algorithms, called structural embeddings, learn representations of the local graph structure around each node. Structural embedding techniques are often used to identify what roles nodes play in their local environment. These algorithms (both classical and structural) have quickly became an intensely researched topics in recent years; see, for example, [8] or a recent book [25], and the list of potential applications constantly increases. After reducing the dimension via node embeddings, node classification can be done more efficiently compared to extracting graph features and using the original network to identify synchronized behaviour. On the other hand, synchronized behaviour should create similar network structure around the involved nodes and so should be captured by the embedding. Such group of nodes may be then potentially extracted (even in an unsupervised way) by some machine learning tools such as DBSCAN that are able to identify dense regions of the embedded space. Some embedding algorithms not only capture local properties of graphs but also try to pay attention to global structure and different roles the nodes play within the network [14, 39] which might carry more predictive power than local **GF**. Additional benefit of such approach, in comparison to using **GF**, is that features are identified automatically in an unsupervised way by the algorithm, as opposed to having to identify them manually by the analyst. Finally, embeddings seem to be less sensitive to sampling techniques and so they might be used as a foundation for more robust classification algorithms. There are many different node embedding algorithms considered in the literature. Additionally these embeddings have many hyperparameters, of which a common among all embedding approaches is their target dimension. Although embeddings were considered for various tasks in the earlier literature, an analysis of how useful they can be for bot detection remains an open field that we investigate in this paper. To answer this question we report on predictive power of **NLP**, **P**, **GF**, and **EMB** features. This investigation also allows us to compare classical and structural embeddings to find which of them are more useful for this task. Additionally, we check how target embedding dimension hyperparameter affects their predictive power.

## Research motivation and goals

As we have highlighted in the introduction, there are many aspects of a user data that one could leverage in building models for identifying whether a user is bot or non-bot. In this work, we categorize these features into four groups. The first are features captured from user profile data and features derived using natural language models from user tweets (**NLP** and **P**). The second are simple graph features, computed for nodes like degree or eigenvalue centrality (denoted **GF**). The third are features extracted from the user’s social network using embedding algorithms (**EMB**). **EMB** features are further broken down into classical and structural embeddings. We build bot classification models using various combinations of the above feature sets, for two different datasets (as will be highlighted in the coming sections). It is important to note that our focus is not on identifying whether bot accounts are malicious or benign. Also, we are not introducing a new bot classification model, rather our aim is to compare the predictive power of these feature sets (**NLP**, **P**, **GF** and **EMB**) with the focus on node (classical) and structural features extracted using embedding algorithms as a new and novel source of predictive features. More specifically, we compare the predictive power of structural versus classical embeddings and show that bot accounts on Twitter often form local social structures, which can be captured by structural embedding techniques.

The main contributions of our paper are as follows:

- By analyzing the performance of bot detection models, using various combinations of feature sets we show that all three feature sets (**NLP**, **P**, **GF** and **EMB**) have predictive power for identifying bot accounts.
- Addition of classical and structural features enhances the performance of bot detection models, hinting at the fact that there are clues for detecting bot accounts in the graph features extracted from the structure of the social network of the users that are not captured by other types of features.
- By analyzing six different embedding algorithms (**EMB**) and comparing their performance to human engineered features (**GF**), we show that embedding algorithms can capture features having predictive power in an unsupervised way that is difficult to design manually.
- Using two different Twitter datasets, we show that features extracted using structural embedding techniques have higher predictive power as compared to features learned using classical embedding techniques.
- We perform dimensionality analysis on both structural and classical embeddings and show that increasing dimensionality of embeddings does not bring much value added. Already low-dimensional embeddings are useful for bot detection.
- Lastly, we perform stability analysis against our embedding features and show that models built using embedding algorithms can be resistant to the addition of noise in the underlying network.

Finally, let us stress that despite the fact that these results are optimistic and show a potential of algorithms based on graph embeddings, this is an early stage of research in this direction. We finish the paper with a discussion of future work that will deepen our understanding of the power (as well as potential issues) of embedding algorithms.

## Related work

In this section, we provide a brief overview of various studies focusing on bot detection algorithms as well as feature sets used for building such models. There are numerous studies focusing on feature engineering and feature extraction from user information on social media networks such as Twitter [18, 27, 30, 33, 45]. For example, in their work Minnich et al. [33] categorize the feature sets used for detecting bots into the following categories: metadata-based features, content-based features, temporal-based features, and network-based features. In this work, the authors mention the importance of including information about the social network of bot accounts such as the number of followers captured by node out-degrees. Similar feature sets were used by Lee et al. [26], where the authors focus on extracting features from user generated information such as tweets and profile data, in addition to first degree graph features such as node degree. Indeed, many of the research efforts on extracting network features are focused on first degree features that can be mapped to real-world metrics such as the number of followers or friends, in addition to network of users who directly interact with user’s tweets via liking or re-sharing them. As we will show in our work, higher order network features such as the network structure of a user’s followers (follower’s of your followers) can have additional predictive power when comes to building bot classification algorithms. There are also a number of studies focused on using node and graph embeddings as features (in addition to other types of features mentioned above) for building bot detection algorithm [3, 4, 22, 29, 38]. For example, Alkulaib et al. [4] build a bot detection technique using the anomalous properties of certain nodes in the graph. The authors use a graph transformer as a self-attention encoder to learn both node and structural representations of nodes. In another work, Hamdi et al. [22] investigate fake news detection on Twitter using node and graph embeddings. Although the studies mentioned here highlight the fact that embeddings can be used as a source of features with predictive power for building classifiers on the social network, they do not explore the difference between structural versus classical (node) embedding techniques.

Although the majority of the effort has been focused on identifying bots, there has been some recent research that focuses on identifying whether bot accounts are malicious or benign. For example, Mbona et al. [31] use features generated from user information to predict whether a user account is malicious or benign. In their paper, Mbona et al. [31] use similar feature sets such as user information and tweet data for analyzing their models. Other recent research such as Tan et al. [44] work on estimating the number of bots in a given twitter community. In this work, the authors use user data, tweet data in addition to graph data to build models that estimate the number of bots in a various twitter communities. Of course the success of any classification model relies heavily on how the underlying dataset was constructed and the quality of the ground truth labels. In the following section, we will introduce two datasets, which will be used in this work for benchmarking the predictive power of various extracted feature sets.

## Datasets

Developing and evaluating bot detection algorithms relies on the availability of unbiased labeled datasets. Although there are numerous datasets used for building and benchmarking bot detection algorithms, we mainly focus on using two recently curated Twitter datasets by Feng et al. [15] [**TwiBot-20**] and Stella et al. [43] [**Italian Election**]. We recognize that labeled bot-datasets often contain some level of bias, since the real ground-truth is not readily available. In general, labels are identified by careful analysis of humans or by cleverly designed algorithms. Throughout our study, we ensure to stay aware of this fact and highlight any impact this may have on our findings.

In the **TwiBot-20** dataset, the authors focus on building a comprehensive Twitter dataset composed of semantic, property, and neighbourhood information. Here, semantic is the Tweet text generated by the user; property is the information related to user profile such as number of followers and following, and finally, neighbourhood is the network structure of the user. We highlight the features used from this dataset below. To capture a natural representation of the ground-truth Twittersphere, the authors implemented a breadth-first search algorithm, to sample and build the dataset. In this methodology, a user is selected as the root of the tree and subsequent layers are built using the directed follow edges of each user. This process is repeated up to layer 3, creating a sample network with a selected user at its root [15]. The sampling algorithm used by the authors builds a directed graph, where nodes are users and edges are follow relationship. As highlighted by Feng et al., this method of sampling does not focus on any particular topic or pattern and should be a more natural representation of the Twittersphere.

We compile a list of raw features available from the **TwiBot-20** dataset in Table 1. Note that the values for these features are a snapshot captured at the time of sampling. We categorize each feature into three types: Profile, NLP and Graph. The profile features are datapoints available through Twitter’s API, and highlight some properties of each user. As pointed out by Feng et al., the followers and following are randomly selected. We use the raw user Tweets as the input to our NLP feature engineering. We provide more detail in our NLP feature analysis section. Lastly, graph features are build using the raw edge list provided in the **TwiBot-20** dataset. As mentioned before, an edge between two nodes indicated a follow relationship between the nodes. Although the original network provided by Feng et al. is a directed graph, we convert it to undirected graph for our analysis. Lastly, we note that the profile feature *verified* is excluded from the bot classification process. This is done for two main reasons. Firstly, most users accounts are not subject Twitter’s verification process, where an account is confirmed to be owned by the user it claims to be. This process would inherently exclude bots from being verified. Secondly, due to the nature of the verification process, this feature could introduce bias for any classifier, thus making the discovery other meaningful features more difficult.

**Table 1:** Feature list for **TwiBot-20** dataset

Type	Feature	Description
Profile	*protected*	When true, indicates that this user has chosen to protect their Tweets.
Profile	$followers\_count$	The number of followers this account currently has.
Profile	$friends\_count$	The number of users this account was following (AKA their “followings”) at the time.
Profile	$listed\_count$	The number of public lists that this user is a member of.
Profile	$favourites\_count$	The number of Tweets this user has liked in the account’s lifetime.
Profile	$statuses\_count$	The number of Tweets (including retweets) issued by the user.
Profile	$geo\_enabled$	When true, indicates that this user enables geolocation (deprecated in today’s API).
Profile	$default\_profile$	When true, indicates that the user has not altered the theme or background of their user profile.
Profile	$default\_profile\_image$	When true, indicates that the user has not uploaded their own profile image and a default image is used instead.
Profile	*business*	User interest domain: business (from [15]).
Profile	*entertainment*	User interest domain: entertainment (from [15]).
Profile	*politics*	User interest domain: politics (from [15]).
Profile	*sports*	User interest domain: sports (from [15]).
Profile	*verified*	True, if the user if verified by Twitter. This is the blue check-mark indicator in user’s profile.
NLP	$raw\_tweets$	The raw recent 200 tweets for each root user.
Graph	graph*	Degree Centrality, Strength, Eigen Centrality, Closeness, Harmonic Centrality, Betweenness, Authority, Hub Score, Constraint, Coreness, Eccentricity, PageRank

*Note that the graph features are calculated by us and not available in the original dataset

In the **Italian Election** dataset, Setlla et al. [43] aim to investigate the online social interactions during a 2018 Italian election and how it helps to understand the political landscape. In their work, the authors study relationship between real users and bots, using the Twitter network. Unlike the **TwiBot-20** dataset, the authors build a sample of the social network by focusing on tweets containing a list of political topics; such as “#ItalyElection2018”, “#voto”, etc. The sampling technique used by Setlla et al. results in a network with a vastly different graph topology than that created by Feng et al. By sampling the TwitterSphere based on topics, the authors created a dataset in which nodes are users and edges represent interactions between users, such as retweets or mentions. Although this makes it difficult to compare the performance of bot detection algorithm between these two datasets, having diversity in how a social network is constructed helps us understand how bots manifest themselves within a network. The **Italian Election** dataset also contains labels indicating if a user is identified as a bot or not. As described by the authors, the bot/not-bot labels were generated by using an a classifier trained using Twitter user’s profile information [43]. Although the original dataset used by Setlla et al. [43] contains user profile and raw Tweet data, in this work we only have access to the network data and thus we can only focus on features extracted from the underlying network structure. Similar to the **TwiBot-20** network, the **Italian Election** graph is directed, with edges pointing from users who interact with other user’s content. We also convert the **Italian Election** graph into an undirected graph for the purpose of our study.

We summarize some high-level statistics of both networks in Table 2. It is important to note that we apply additional data cleansing and filtering to provided dataset. For example, we run our analysis on the largest component of each graph, and convert both graphs into undirected networks. The reason for converting these graphs to undirected networks is that some embedding algorithms only take undirected graphs as input. Using undirected network ensures that comparison between the performance of each embedding is fair. It is important to note however that by converting graphs from directed to undirected we lose some (potentially predictive) information. Lastly, we note that the sampling technique used to construct the above two networks has potential impact on the level predictive information captured by featured built using node/graph statistics and embedding algorithms. One could construct a social network based a variety of information, for example edges could represent follow/friend relationship or retweet or like relationship. Nodes themselves could represent users or tweets.

**Table 2:** Graph statistics for the **Twibot-20** and **Italian Election** datasets

Metric	**Twibot-20**	**Italian Election**
Number of Nodes	156,115	12,404
Number of Edges	166,764	21,029
Size of Largest Connected Component	142,280	7,807
Number of Components	646	586
Avg Degree (Bots)	20.13	3.59
Avg Degree (Not-Bot)	20.38	3.28
Number of Isolated Nodes	0	0
Avg Community Size [Louvain]	172.3	19.7
Number of Communities [Louvain]	906	631
Modularity [Undirected]	0.945	0.796

## Profile and NLP features

**Fig. 1:**
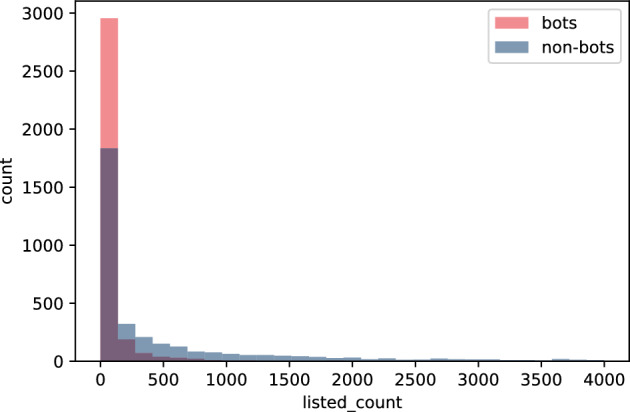
Histogram of $listed\_count$, number of public lists that users are a member of, for bots and non-bots

In this section we focus on features extracted from user’s profile information and their tweets. We perform feature engineering, specially on the raw tweets using various NLP techniques. Since we only have profile and tweet data from the **Twibot-20**, our analysis is centered around this dataset. To maximize their impact on a social network, bots aim to mimic real-user behaviour. To this end, bots aim to create accounts and content that seem natural, such that it was generated by a real user. An example of such actions could include following other users, tweeting about relevant topics and engaging in conversations. Despite their effort, as we will discuss in this section, bots often leave behind signs that allow us to distinguish them from non-bots. Starting with the profile Twitter API data named in "Datasets" section, the number of public lists that a user is a member of, $listed\_count$, is strikingly different for the two groups (bots vs. non-bots)—see Fig. 1 and Table 3. It is a measure of user’s popularity, and it turns out that humans tend to be added to Twitter lists by other users of the platform more often than bots are. It means that in general Twitter users value human-generated tweets and intuitively prefer this type of content.

**Table 3:** Statistics for the profile features belonging to the **Twibot-20**’s bot and non-bot accounts

Feature	KS p-value	Bot			Non-Bot		
		Mean	STD	Median	Mean	STD	Median
$friends\_count$	5.069e-25	5,084.816	24,215.345	839.0	8,732.032	87,998.503	614.0
$followers\_count$	9.999e-01	20,735.2	182,269.0	724.0	1,490,368.1	6,465,366.5	27,322.0
$listed\_count$	9.999e-01	112.172	642.845	4.0	3,366.707	14,049.919	212.0
$statuses\_count$	8.457e-97	15,546.791	51,880.579	1,971.0	29,356.907	110,080.653	6,577.0
$links\_per\_tweet$	9.075e-08	0.517	0.376	0.420	0.531	0.359	0.464
$mentions\_per\_tweet$	1.049e-63	1.164	1.07	1.11	0.961	0.6	0.940
$av\_tweet\_len$	1.763e-18	112.831	39.384	109.5	114.993	45.373	109.98
$std\_tweet\_len$	9.151e-64	49.543	21.826	46.883	54.8	19.039	54.627
$no\_langs$	3.759e-28	4.2	3.079	3.0	5.083	3.804	4.0
$perc\_en$	8.082e-79	0.916	0.182	0.975	0.77	0.345	0.955
$no\_odd\_langs$	9.992e-25	3.11	3.06	2.0	3.897	3.723	3.0
$perc\_legit$	1.253e-18	0.964	0.045	0.98	0.954	0.053	0.97
$av\_sent$	1.122e-34	-0.371	0.342	-0.414	-0.316	0.378	-0.331
$std\_sent$	4.457e-27	0.788	0.224	0.859	0.785	0.259	0.886
$positive\_sent\_perc$	2.538e-35	0.311	0.174	0.289	0.339	0.193	0.333
$links\_no$	1.6e-33	84.432	73.847	66.0	98.386	74.241	83.0
$mentions\_no$	9.141e-26	196.124	210.408	194.0	174.355	124.118	172.0
$tweets\_no$	1.031e-90	163.465	66.833	199.0	178.841	54.876	200.0

**Fig. 2:**
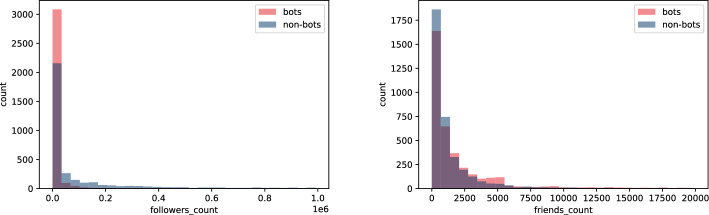
Histogram of $followers\_count$, number of users that follow the account (left), and $friends\_count$, number of user’s followings (right), for bots and non-bots

It is worth noting the difference between the number of users that follow them ($followers\_count$) and the number of accounts the user follows ($friends\_count$)—see Fig. 2 and Table 3. There is a clear asymmetry here. In general, humans follow less users and get followed more than bots do. The reason behind this could be that bots become friends with many users in order to seem more legitimate and, at the same time, human users are less interested in bot-generated content than the one created by humans.

In addition to the original profile data from the Twitter API we listed in "Datasets" section, we have extracted a number of features from user tweets in the **Twibot-20** dataset. This was done by sampling each user’s tweets and running NLP feature extraction on them. For language detection we utilized fastText python module [6], whereas for the sentiment analysis (only for the English tweets) we used HuggingFace, transformer pre-trained model [46]. Here is the list of extracted features:

- $links\_no$ - total number of hyperlinks in user tweets
- $mentions\_no$ - total number of references to other users in user tweets
- $tweets\_no$ - total number of tweets generated by user
- $links\_per\_tweet$ - average number of hyperlinks per tweet$^{1}$
- $mentions\_per\_tweet$ - average number of references to other users per tweet^1^
- $av\_tweet\_len$ - average tweet length (in characters)
- $std\_tweet\_len$ - standard deviation of tweet length
- $no\_langs$ - number of dominant languages used in tweets (it is assumed that each tweet has exactly one dominant language)
- $perc\_en$ - percentage of tweets written in English
- $no\_odd\_langs$ - number of languages present in less than 10% of tweets
- $perc\_legit$ - percentage of tweets written in languages present in more than 10% of tweets
- $av\_sent$ - average sentiment score (using the scores of the dominant labels)
- $std\_sent$ - standard deviation of sentiment score (using the scores of the dominant labels, e.g., taking $-$0.9 for 0.9 score for negative sentiment)
- $positive\_sent\_perc$ - percentage of English tweets with positive sentiment assigned

**Fig. 3:**
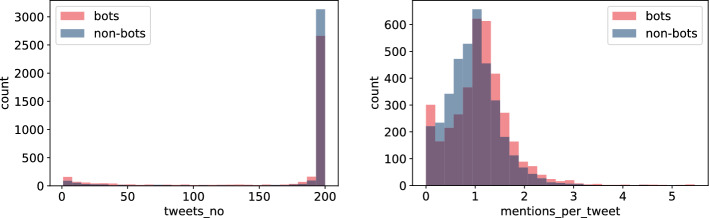
Histogram of $tweets\_no$, total number of tweets (left), and $mentions\_per\_tweet$, average number of references to other users per tweet (right), for bots and non-bots

Based on exploratory data analysis, there are some noticeable differences between bots and non-bots. First of all, as reported in [15], bots in **Twibot-20** generate fewer tweets than humans do—see Fig. 3 (left). This is quite surprising and in contrast with earlier findings reported in [36]. This difference may be attributed to the fact that bots change behaviour with time, they are constantly getting more clever. Currently, they interact with the system only to achieve a very specific goal and often disappear shortly after, generating fewer tweets in total. This indicates that **NLP** approach cannot be easily generalized and might require constant re-training.

It seems that bots tag other users more frequently than humans do—see Fig. 3 (right). The reason behind might be that some types of bots do not produce much of their own content but, instead, tag many different users to generate their attention and hope for a potential link click. This is reflected in $mentions\_per\_tweet$ (the average number of references to other users per tweet) depicted in the figure but also in $mentions\_no$, its cumulative counterpart. We used the former value in further analyses.

**Fig. 4:**
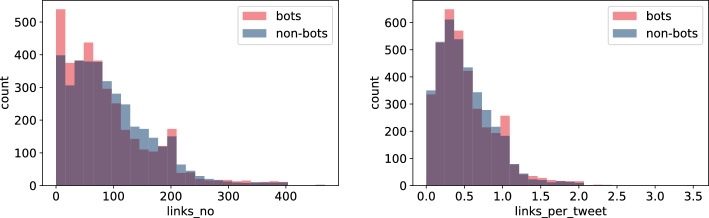
Histogram of $links\_no$, the total number of hyperlinks (left), and $links\_per\_tweet$, the number of hyperlinks per tweet (right), for bots and non-bots

Perhaps surprisingly, Fig. 4 suggests that the same reasoning cannot be as easily applied to $links\_no$ and its counterpart $links\_per\_tweet$, focusing on the number of hyperlinks generated by users. The two charts are more ambiguous. For $links\_no$, it seems that humans include more hyperlinks in total than bots do. The lowest values of this feature (less than 80) are dominated by bots and the largest ones are non-conclusive (interchanging between bots and humans dominance). However, if one looks at the per tweet counterpart, the largest values in the distribution are visibly assigned to bots more often than to humans. Bots achieve the extreme values of $links\_per\_tweet$ about twice as frequently as non-bots even though the direction was not obvious for the absolute value—the conclusion is that humans generate more hyperlinks because they generate more tweets. For this reason, we will exclude $links\_no$ from the analysis and work using per tweet features instead.

**Fig. 5:**
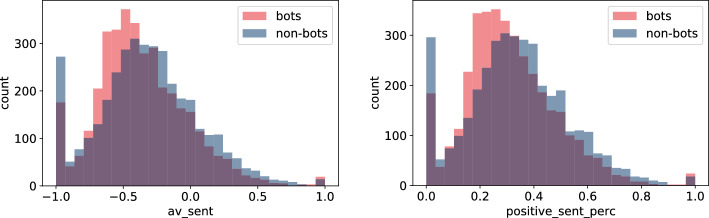
Histogram of $av\_sent$, average sentiment score (left), and $positive\_sent\_perc$, percentage of positive tweets (right), for bots and non-bots

In both examples above, we removed two features that are highly correlated, without affecting the quality of the model. Of course, one does not need to do it and let the classifier to deal with this situation. Removing redundant features is, however, a good practice and the main reason to perform EDI. It reduces the dimension of the problem and so improves scalability. It is another evidence that **NLP** approach requires supervision of domain experts and careful investigation.

One of the most noticeable differences seems to be the one in the sentiment of the posts. In Fig. 5 there is a visible mismatch between bots and non-bots, both in terms of sentiment score (left) and sentiment label (right). While for both groups the histograms are right-skewed, tweets posted by non-humans in general tend to be more negative than those created by real people. Bots may be present on social media platforms to lead a campaign against some product, company or political fraction and their actions are deterministic, not affected by external sources. On the other hand, humans seem to react more strongly to negative stimuli, that is, we believe in negative opinions more so than praise [41]. It may explain the observed phenomenon.

**Fig. 6:**
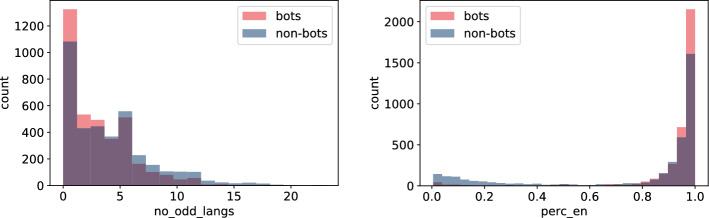
Histogram of $no\_odd\_langs$, number of languages present in less than 10% of user tweets (left), and $perc\_en$, percentage of tweets written in English (right) for bots and non-bots

As one would expect, the way tweets are written seems to be different linguistically between bots and non-bots. Language detection performed on tweets suggests that humans may write in a more convoluted way in which case the fastText model fails to detect tweet language properly. In Fig. 6 (left) we present the number of languages that the user used in less than 10% of their tweets, i.e. $no\_odd\_langs$. Larger fraction of bots use zero or only one rare language. The same result holds for $no\_langs$ and $perc\_legit$. Moreover, bots seem to use mostly English as can be observed in Fig. 6 (right). There are only a few bots in the dataset for which the percentage of tweets written in English ($perc\_en$) is low.

With recent advances in Transformer models, computer generated text is becoming evermore human like. The current state-of-the-art is the OpenAI’s GPT-3 [7], a generative model for NLP tasks with 175 billion parameters! GPT-3 has been demonstrated to be effective on a variety of few-shot tasks: due to its extensive pre-training and size, it is able to learn rapidly from very few training examples. It generates texts that are nearly indistinguishable from human-written texts: Humans correctly distinguished GPT-3 generated texts from real human texts approximately 52% of the time, which is not significantly higher than a random chance [7]. For more details on GPT-3 and other related topics we direct the reader to, for example, a recent survey [30].

**Fig. 7:**
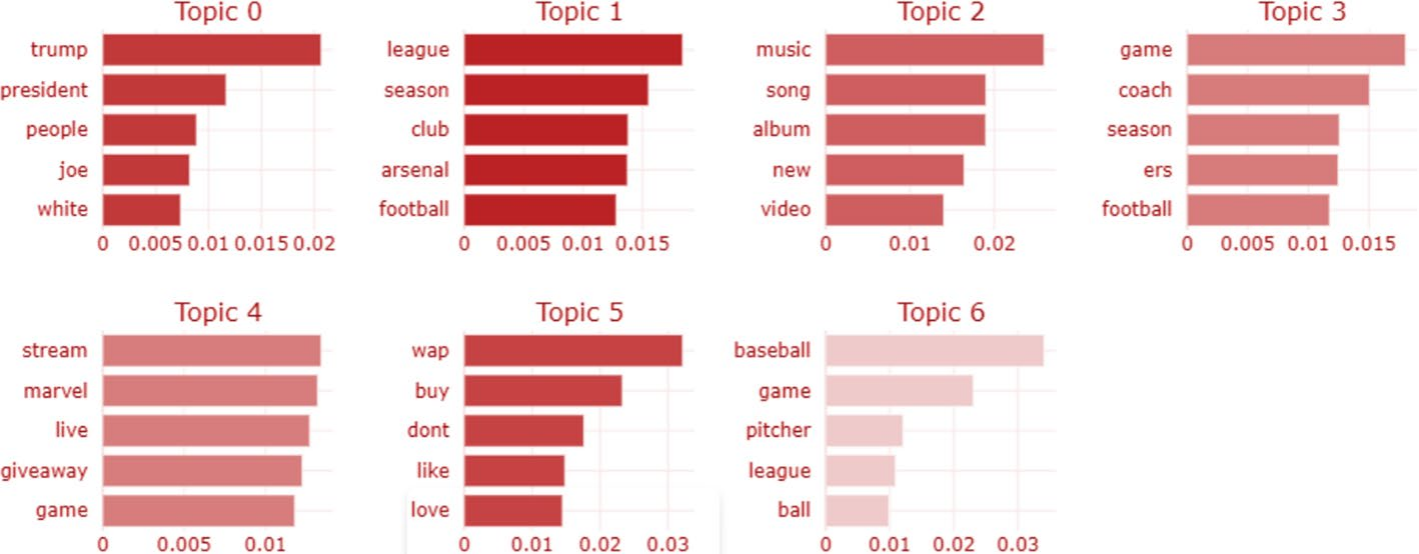
Topic scores for tweets made by bots

**Fig. 8:**
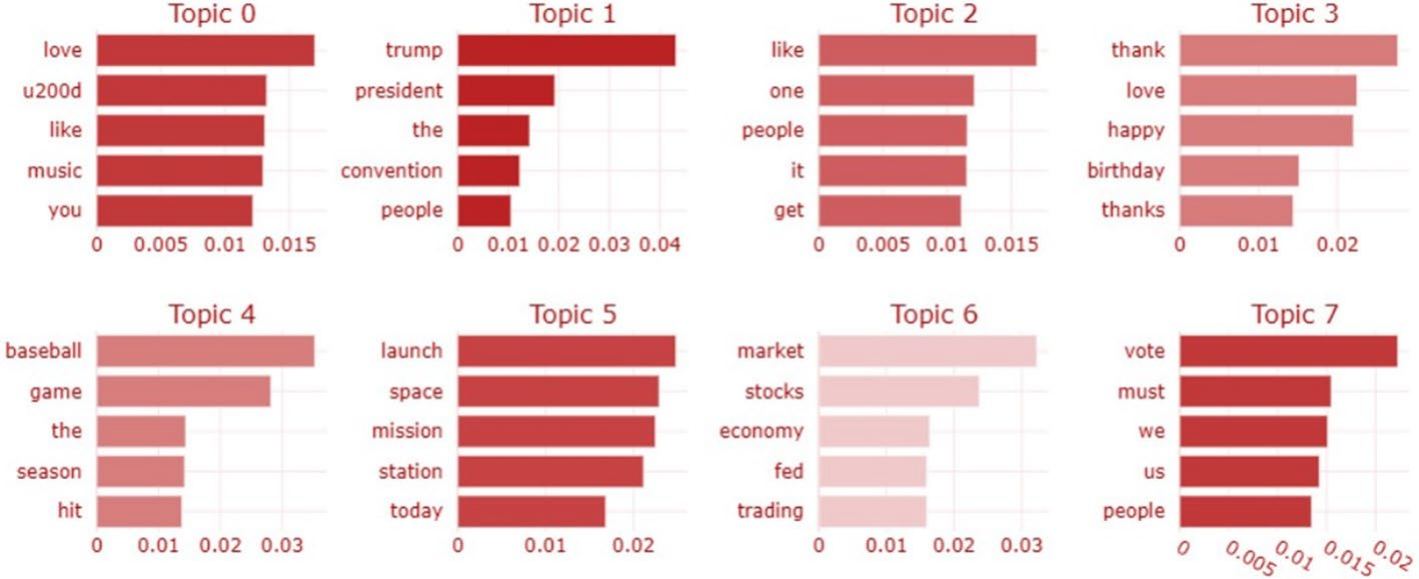
Topic scores for tweets made by non-bots

Another potential source of information for identifying bot accounts lies within the raw tweet text produced by each user. To this end, we perform topic modeling using BERTopic (BERT for Topic Modelling) [20]. BERTopic is a topic modelling technique that uses transformers and the c-TF-IDF to produce dense clusters that allow for clearly understandable topics while maintaining key phrases in the topic descriptions. This is done to gain insight into difference in the type of topics bot and non-bot account focus on while interacting with other users. In Figs. 7 and 8 we highlight the score of various topics used by bot and non-bot accounts. The graph represents relative c-TF-IDF scores between and within topics. The darker shades basically means that the tweets in which the words from a particular topic appeared are strongly related with each other. Topics extracted here are tokenized and used in the bot classification models, as we will highlight in later sections. Note that topics for bots and non bots are not the same (e.g. topic 0 for bots is roughly topic 1 for non bots).

## Graph derived features

We showed in "Profile and NLP features" section that there are statistically significant differences in **NLP** and **P** features between bot and non-bot accounts. Another potentially independent source for extracting features is rooted in the way users/bots interact with others in the network. One can capture this information by analyzing various graph properties derived from the underlying social-network. This can be done in two ways. One, by carefully designing statistical features of the nodes. Second, using unsupervised methods to learn node and structural representations of the nodes. In this section, we provide a detailed analysis of node feature engineering in addition to features extracted using various embedding techniques.

### Node features

In this section, we build node features derived from the underlying network structure using both **TwiBot-20** and **Italian Election** datasets. For extracting features we use NetworkX as well as igraph python packages depending on the efficiency of the corresponding algorithms. Here is the list of extracted node-features that were computed for all nodes. For detailed definition we direct the reader to, for example, [25] or any other textbook on network science. -

- $degree\_centrality$ - degree (the number of edges the vertex has)
- *strength* - minimum ratio of edges removed/components created during graph decomposition process
- $eigen\_centrality$ - eigenvector centrality, a measure of the importance of the vertex (using relative scores)
- *closeness* - closeness centrality, a measure of the importance of the vertex calculated using the sum of the length of the shortest path between the vertex and other vertices
- $harmonic\_centrality$ - harmonic centrality (another variant of closeness centrality, calculated similarly)
- *betweenness* - betweenness centrality, a measure of the importance of the vertex calculated using number of shortest paths that pass through the node
- *authority* - authority score, sum of the scaled hub values that have edge to the given node
- $hub\_score$ - hub score, sum of the scaled authority values of the nodes it has edge to
- *constraint* - Burt’s constraint, an index that measures the extent to which a person’s contacts are redundant
- *coreness* - coreness (unique value of *k* such that a node belongs to the *k*-core but not to the $(k+1)$-core)
- *eccentricity* - eccentricity (the maximum distance from a given node to other nodes)
- *pagerank* - another way of measuring node importance - invented by Google Search to rank web pages in Google search engine output

In addition to the above list of features, we compute average, standard deviation, minimum and maximum of every feature for the neighbouring nodes of each vertex. A full list of these features is given in Tables 6 and 7.

**Fig. 9:**
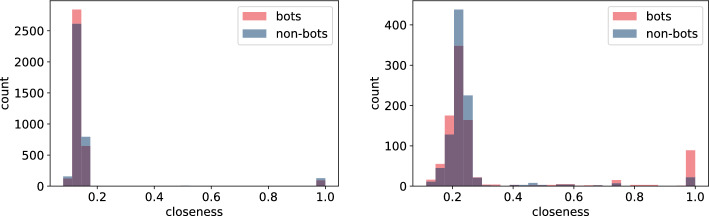
Histogram of closeness centrality measure for bots and non-bot users’ neighbours for **TwiBot-20** (left) and **Italian Election** dataset (right)

**Fig. 10:**
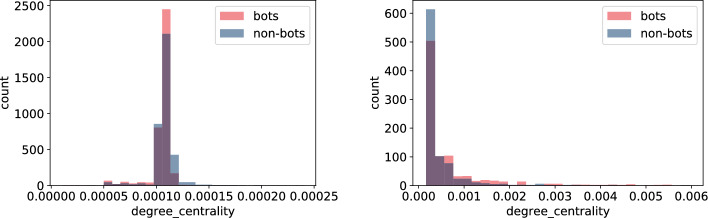
Histogram of closeness centrality measure for bots and non-bot users’ neighbours for **TwiBot-20** (left) and **Italian Election** dataset (right)

**Fig. 11:**
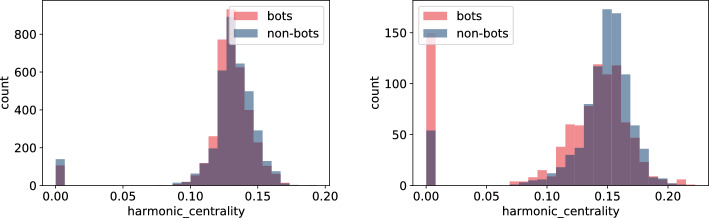
Histogram of harmonic centrality measure for bots and non-bot users’ neighbours for **TwiBot-20** (left) and **Italian Election** dataset (right)

**Fig. 12:**
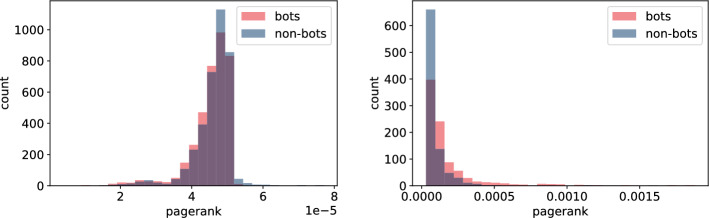
Histogram of pagerank measure for bots and non-bot users’ neighbours for **TwiBot-20** (left) and **Italian Election** dataset (right)

As we highlighted in "Datasets" section, there are major differences in how the **TwiBot-20** and **Italian Election** datasets were constructed. Firstly, the underlying network constructed in the **TwiBot-20** captures follower-following relationship, while the network in the **Italian Election** dataset represents interactions between users. Secondly, the sampling technique used in the **TwiBot-20** dataset results in much more uniform graph topology since at each sampling layer a fix number of nodes (followers) were sampled. This is in contrast to the **Italian Election** dataset, were nodes were more randomly sampled. The difference in the network topology between these two datasets is reflected in the values captured using the node-features, as shown in Figs. 9, 10, 11, and 12. This is indeed an important observation, since one could extract more meaningful node-features by resampling the same underlying graph using different techniques.

Secondly, the features which calculation did not involved neighbours (Figs. 9, 10, 11, and 12) indicate only slight differences between bots and non-bots, both in terms of feature count and magnitude of discrepancies. Nevertheless, in the case of harmonic and closeness centrality (Figs. 11 and 9) the difference is more visible on the **Italian Election** dataset: bots seem to be more likely to take extreme values. Regarding the **TwiBot-20** dataset, the discrepancies between bot and non-bots are less visible, but all closeness, degree, harmonic centrality, and pagerank measure distributions seem to be more left-skewed for non-bots. (Figs. 9, 10, 11, and 12).

**Fig. 13:**
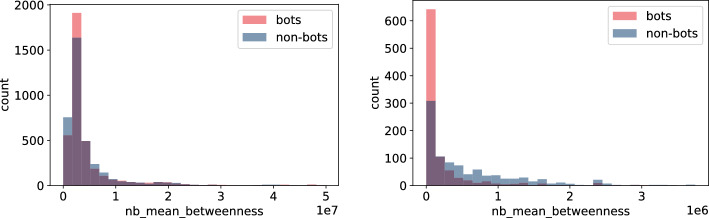
Histogram of neighbours’ mean betweenness measures for bots and non-bot users’ neighbours for **TwiBot-20** (left) and **Italian Election** dataset (right)

**Fig. 14:**
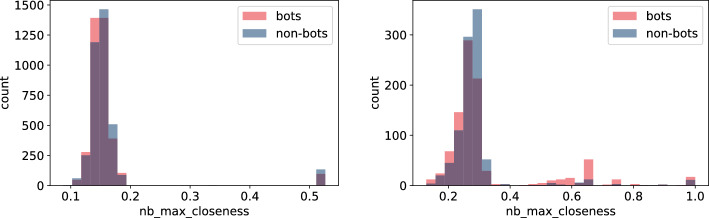
Histogram of neighbours’ max closeness measures for bots and non-bot users’ neighbours for **TwiBot-20** (left) and **Italian Election** dataset (right)

**Fig. 15:**
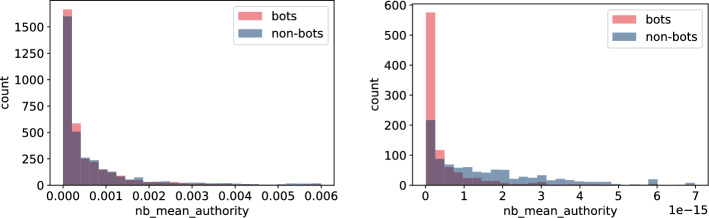
Histogram of neighbours’ mean authority measures for bots and non-bot users’ neighbours for **TwiBot-20** (left) and **Italian Election** dataset (right)

**Fig. 16:**
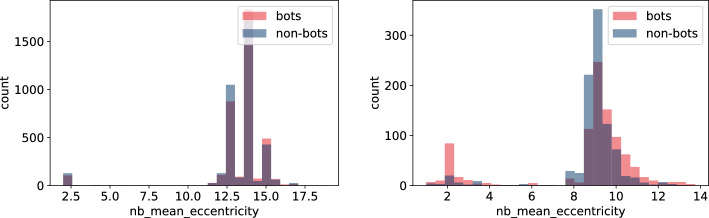
Histogram of neighbours’ mean eccentricity measures for bots and non-bot users’ neighbours for **TwiBot-20** (left) and **Italian Election** dataset (right)

Figures 13, 14, 15, and 16 reveal that discrepancies between bot and non-bots groups are more visible on the distributions of features involving particular nodes’ neighbours’ during calculation. Similarly to the previous group of characteristics, differences are more visible on the **Italian Election** data and again, values in this dataset seem to have lower variance (Figs. 13, 14, and 15) or variance among groups (Fig. 16). In particular, values for bots’ features seem to have even lower standard deviation (Figs. 14 and 16), which may be an indicator of the fact that bots constitute a homogenous group. Nevertheless, as different conclusions may be drawn on the basis of **TwiBot-20** dataset (Figs. 13, 14, 15, and 16), so this observation may be attributed to different sampling or annotating method.

The fact that node features constructed on the basis of data about vertices’ neighbours may help in explaining being bot versus non-bot (at least more than pure node features) indicates the purposefulness of node embeddings usage. However, as this assumption is based solely on graphical analysis, one may be interested in modelling the relationship of node features and “being a bot”. This is done in the following sections.

### Classical and structural embeddings

There are over 100 algorithms proposed in the literature for classical and structural embeddings which are based on various approaches such as random walks, linear algebra, and deep learning [19, 25]. Moreover, many of these algorithms have various parameters that can be carefully tuned to generate embeddings in some multidimensional spaces, possibly in different dimensions. In this paper, we typically set all parameters but the dimension to the default values recommended by their authors. Once parameters are fixed, the algorithms learn the embedding in an unsupervised way. Having said that, some algorithms are randomized and so the outcome might vary. For our experiments, we selected 6 popular algorithms that span different families and includes both node as well as structural embeddings.

The first two algorithms, Deep Walk [37] and Node2Vec [21], are based on random walks performed on the graph. This approach was successfully used in NLP; for example the Word2Vec algorithm [32] is based on the assumption that “words are known by the company they keep”. For a given word, embedding is achieved by looking at words appearing close to each other as defined by context windows (groups of consecutive words). For graphs, the nodes play the role of words and “sentences” are constructed via random walks. The exact procedure how one performs such random walks differs for the two algorithms we selected.

In the Deep Walk algorithm, the family of walks is sampled by performing random walks on graph *G*, typically between 32 and 64 per node, and for some fixed length. The walks are then used as sentences. For each node $v_i$, the algorithm tries to find an embedding $e_i$ of $v_i$ that maximizes the approximated likelihood of observing the nodes in its context windows obtained from generated walks, assuming independence of observations.

In Node2Vec, biased random walks are defined via two main parameters. The *return parameter* (*p*) controls the likelihood of immediately revisiting a node in the random walk. Setting it to a high value ensures that we are less likely to sample an already-visited node in the following two steps. The *in-out parameter* (*q*) allows the search to differentiate between inward and outward nodes so we can smoothly interpolate between breadth-first-search (BFS) and depth-first search (DFS) exploration.

The above algorithms primarily capture proximity among the nodes, nodes that are close to one another in the network are embedded together. But proximity among the nodes does not always imply similarity, as in the specific application we consider in this paper, bot detection. A role the nodes play within the network depends more on the structure of the network around them more than the distance between them. (See [40] for a survey on roles.) The next four algorithms aim to create embeddings that capture structural properties of the network.

The first algorithm from this family, Role2Vec [1], generalizes the above techniques based on traditional random walks. To capture whether two nodes have the same role within the network, the notion of attributed random walks is introduced which is not tided to node identity but is instead using a function that maps a node attribute vector to a role. As a result, the algorithm learns associations among subsets of nodes (that is, roles) instead of properties of individual nodes.

RolX [23] is another approach to explicitly identify the role of nodes using exclusively the network structure. This algorithm is based on enumerating various structural features for nodes in an unsupervised way, and finding the most suited basis vector for this joint feature space. Then, the algorithm assigns every node with a distribution over the identified roles (basis), allowing for mixed membership across the roles.

The next algorithm, Struc2Vec [39] uses a hierarchy to measure node similarity at different scales. As a result, it constructs a multilayer graph to encode structural similarities and generate structural context for nodes. This hierarchical view is useful as it provides a sequence of more restricted notions of what it means to be structurally similar. At the bottom of the hierarchy, similarity between nodes depend exclusively on their degrees whereas at the top of the hierarchy similarity depends on the entire network.

The last algorithm we tested, GraphWave [14] uses techniques from graph signal processing. It learns structural embeddings by propagating a unit of energy from a given node and characterizes its neighbouring topology based on the response of the network to this probe. The runtime of this algorithms scales linearly with the number of edges.

As mentioned earlier, we fix most of the hyperparamters of each algorithm to their default value, and only adjust the embedding dimension. Of course, it is important to note that it is possible to optimize the outcome of each algorithm by searching for the ideal set of parameters for the task at hand, however our goal is not to optimize for the best metrics, but rather learn whether embeddings can help us in identifying bots in a social network.

## Bot classification

Thus far, we have focused on engineering and analyzing features built using user-profile, NLP, node-features and embeddings. In effort to understand the predictability of these features in identifying bot accounts, we train and test various classification models using the **TiwBot-20** and **Italian Election** datasets. Since the underlying data for these datasets are different, we divide our analysis into two section accordingly, focusing on each dataset separately. In both cases, datasets are sampled such that bot/non-bot classes are balanced (50/50). Furthermore, we use a 80/20 split for the train/test datasets. A 5-fold cross-validation process is then used to arrive the best performing model and avoid any overfitting. All metrics are then computed using the test-set.

### Bot detection using TwiBot-20 dataset

In this section, we use **NLP**, **P**, **GF** and **EMB** features to build a bot classification model. Our goal is not to optimize for the best performing model, but rather understand the predictive power of each feature-set. We build five models using various combinations of feature-sets. A summary of the performance of each model is highlighted in Table 4. As shown, the best performing model (based on accuracy) is the one trained on all features combined, achieving an accuracy of $81.76 \%$. Furthermore, we note that models trained on **EMB** perform slightly better than those trained on **GF** alone. This enforces the fact that unsupervised embedding algorithms have the potential to learn complex and meaningful node features. More importantly, a model built on a combined **GF+EMB** performs better than **GF** and **EMB** separately, hinting that embedding features capture incremental predictive information about the nodes. Lastly, we note that models built using features extracted from the underlying network (**GF** and **EMB**) suffer from the uniform topology of the **TwiBot-20** dataset, as described previously. A different sampling technique could potentially result in a boost in the predictive power of features built using the network structure.

There are various strengths and weaknesses of the embedding algorithms, which we need to address. In terms of strengths, node and structural embedding algorithms are often unsupervised and generalizable techniques that can be applied to any network. This however may not be the case for profile meta-data and NLP features, as they depend on the specifics of the data source. For example, NLP techniques applied to texts written in English may not apply to other languages. Furthermore, it is more challenging for sophisticated bots to mimic the network properties of a real user as it requires the participation of other users in building their social network. On the other hand, it is much easier for bots to use the cutting edge natural language techniques to generate text, indistinguishable from real humans. There are, however, some issues with using embeddings as a new source of feature-set for training bot detection machine learning models. One of the major challenges with some embedding techniques is that they will require us to use the entire, or at least a large portion of the graph, to build node and structural embeddings. This introduces a problem when it comes to large social networks. To overcome this, one could sample the underlying network. Unlike profile metadata and NLP features that only depend on a single user, embeddings rely on the connections between users. This introduces another challenge, which is that one cannot run an embedding algorithm on a single user, but rather requires us to reconstruct the network surrounding that user. Lastly, embedding algorithms are often computationally expensive, as compared to models built on profile data and simple NLP techniques. In the case of large social networks, one often needs to sample the underlying network to arrive a computationally reasonable system to study.

**Fig. 17:**
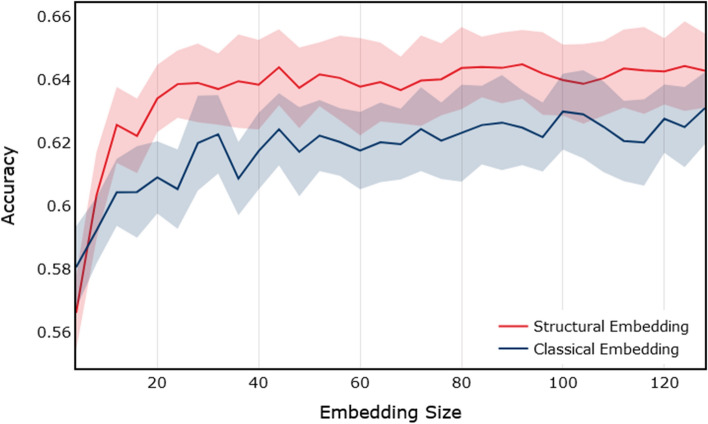
Accuracy as a function of embedding size for structural and classical embeddings. Simulations are performed using the **TwiBot-20** dataset. The solid line corresponds to the mean accuracy for 50 runs, and the band correspond to the standard deviation of the results

Expanding on the analysis of predictive power of embeddings, we focus on the difference in the predictive power between structural versus classical embedding algorithms. Our hypothesis indicates that some bots on social media such as Twitter behave similarly to one another, which can be captured in the role those bots (nodes) play in their local network. This manifests itself in the structure of the local network surrounding a bot. One could capture this using structural embedding algorithms, which are designed to learn representations of the local structure around a given node. On the other hand, classical embedding techniques, which capture proximity within the graph, would learn representations of nodes which should have less predictive power as compared to structural embeddings. To test this hypothesis, we run experiments using both structural and classical embeddings, on the **TwiBot-20** dataset. We embed every node using both classical and structural embedding algorithms, and measure the accuracy of a model designed to classify bots, using only the embeddings as features. The results for these experiments are shown in Fig. 17. We note that in these experiments, structural embeddings are a combination of LSME and Role2Vec, while classical embeddings correspond to a combination of DeepWalk and Node2Vec. For each experiment, we build 50 models, and mean and standard deviation of the accuracy values produced by each model is reported. Lastly, for every experiment, classes (bot and non-bot) are balanced to 50/50 and data is split into train, test and validation sets.

As the first observation, we can see that the performance of the models built using structural embeddings as features is better (by roughly 0.02) than models built using classical embeddings as features. This supports our hypothesis that bots are better identified by the role they play in their local social network, which can be captured using structural embedding techniques. Secondly, we point out that the predictive power in the embeddings (for both classical and structural) increases with the increase in the size of the embeddings, up to a certain size. In our experiments, as seen in Fig. 17, accuracy increases up to embedding size of roughly 35, beyond which the accuracy converges. This result can be interpreted in the following way. The structural and proximity information captured by embedding dimensions greater than 35 do not have any predictive power when it comes to our particular task of bot detection. This information is useful for practitioners in the field, since larger embeddings are computationally more time consuming to train, and one could train smaller embeddings with similar end-result performance. Of course, this will change for every dataset and sampling technique, as the information available for embedding algorithms to capture would be different.

**Table 4:** Performance measure for models trained using **TwiBot-20** dataset

Procedure	Accuracy	Precision	Recall	F1	MCC
**NLP+P**	0.8036	0.8059	0.8675	0.8356	0.5952
**GF**	0.6352	0.6357	0.8563	0.7297	0.2274
**EMB**	0.6481	0.6562	0.8153	0.7271	0.2594
**GF+EMB**	0.6620	0.6599	0.8507	0.7433	0.2905
**NLP+P+GF+EMB**	0.8176	0.8256	0.8657	0.8452	0.6246

**Fig. 18:**
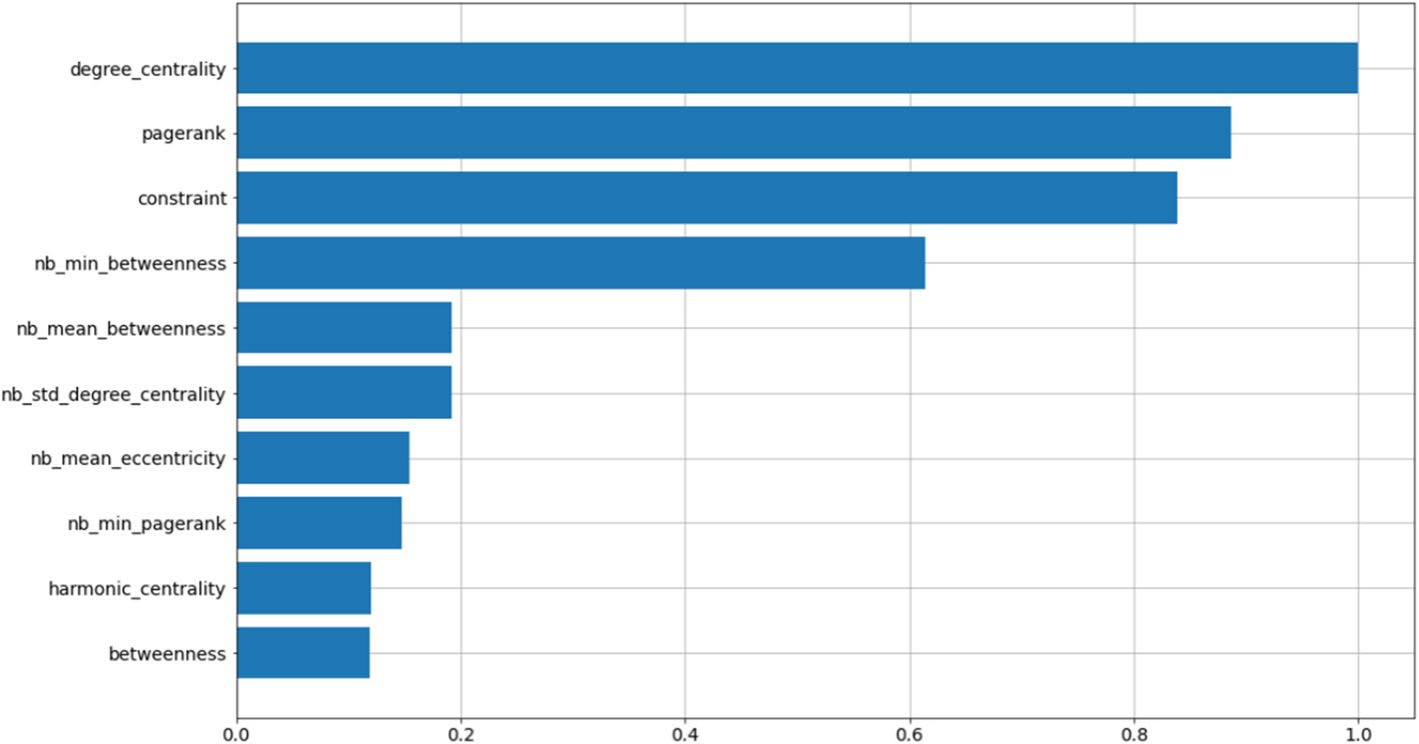
Variable importance for the H2O AutoML leader model built on the basis of node features (exclusively) (**TwiBot-20**)

Next, we use feature importance analysis to explore the contribution of various features in the **GF + NLP + P** feature-sets to the performance of models. Since the embedding algorithms learn continuous representation of nodes in an unsupervised way, it is not easy to reverse engineer what each embedding dimension represents. Starting with feature importance using **GF** feature-set, we highlight in Fig. 18, that top two most predictive features for the **TwiBot-20** datasets are *degree_centrality* and *pagerank*. Given the high overlap in the performance of models built using **GF** and **EMB** datasets, one could postulate that embedding algorithms learn some form of centrality measure about the nodes.

**Fig. 19:**
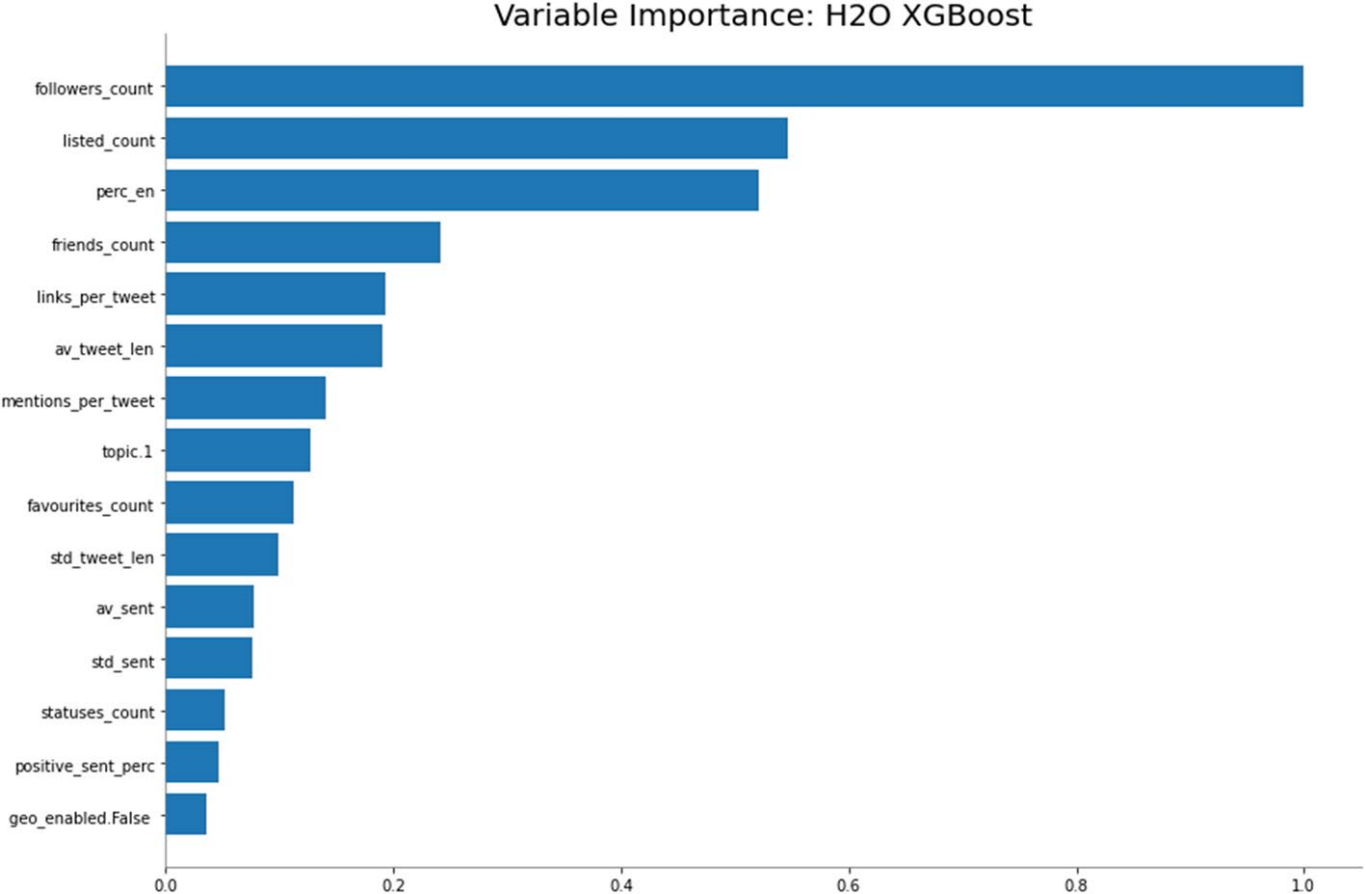
Variable importances for an XGBoost bot classifier on profile and NLP features

Next, we apply similar feature importance analysis to the **NLP** and **P** feature-sets. According to top 20 variable importances in Fig. 19 the set of the top three predictor features are two original Twitter API variables, $followers\_count$, $listed\_count$, and the percentage of English tweets, $perc\_en$ extracted in the course of this study. This feature’s importance has manifested in the basic EDA too. Further behind, we can see three variables with a similar importance, i.e, $friends\_count$, $links\_per\_tweet$ and $av\_tweet\_len$. Quite noticeable impact was noted for user mentions per tweet as well as for sentiment related features.

**Fig. 20:**
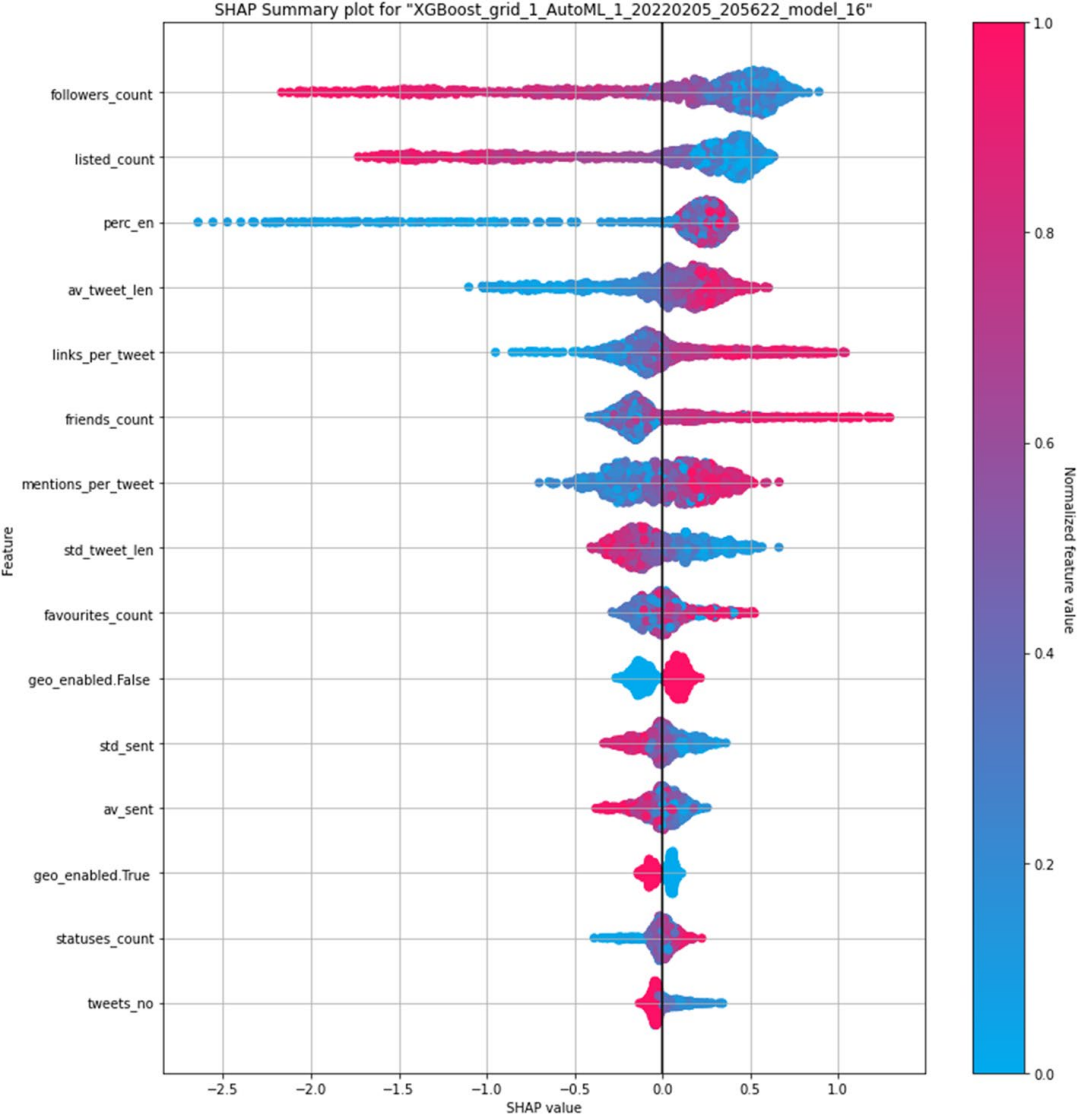
Shapley values for an XGBoost bot classifier on profile and NLP features

A complementary feature importance analysis can be done using Shapley technique. We present Shapley values for the **NLP** and **P** features in Fig. 20. It is confirmed that the more followers users have, the least chance of them being a bot. As in Fig. 2, even though bots are not generally as followed on Twitter as bots are, the relationship is opposite for Twitter friendships—the extreme values for $friends\_count$ are generally associated with bots in the ML classifier as well. Another significant distinction between bots and humans can be found in $listed\_count$. Large number of public lists that this user is a member of is associated with being a human. The percentage of tweets written in English is not as monotonously related to being a bot, but the highest values are characteristic exclusively for bots. These users also seem not to be keen on using geographical tagging when they’re posting (this was an opt-in feature on Twitter). The links and mentions per tweet tend to be higher for the identified bots, even though the initial basic EDA could not detect this tendency clearly for the links feature.

In summary the analysis of the **TwiBot-20** dataset confirms our hypothesis. First, graph embeddings give an additional information for bot detection on top of **NLP**, **P** and **GF** information. Additionally a comparative analysis of **EMB** vs. **GF** shows that embeddings are more useful in this task. Secondly we find that structural embeddings are better than classical embeddings for bot detection. Finally, we learn that embeddings of moderate dimensionality (35 dimensions in our results) are enough and adding more dimensions does not noticeably improve their predictive power.

### Bot detection using Italian-election dataset

In this section, we build and analyze bot classification models using the **Italian Election** dataset. As pointed out earlier, we do not have user profile or tweet data for the **Italian-Election** dataset, and thus all models are built using only the **GF** and **EMB** features. As before, we train our models on balanced datasets containing 50/50 proportion of bot and non-bot examples. Test, train split is kept consistent to 80/20 split. Furthermore, we keep the model architecture and all hyperparameters the same for all runs, to keep performance comparison consistent across all feature sets. We measure a number of metrics when comparing the performance of each feature set, including accuracy, recall, precision and Matthews correlation coefficient (MCC). Since some embeddings such as Node2Vec and DeepWalk are randomized (rely on random walks), we run those algorithms multiple times (sample size 100) and report our findings on the sample.

**Fig. 21:**
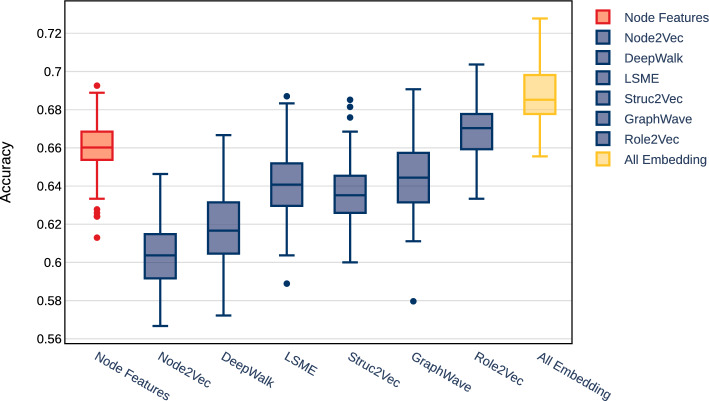
Accuracy for bot classification for the **Italian-Election** dataset. Distributions are over 10 runs of each embedding algorithm and 10 runs of classifier

We summarize the result of our classification analysis in Table 5, highlighting the metrics used for comparing the performance measures. As a reference, we plot the model accuracy for each feature set in Fig. 21. We note that the goal for this analysis was not to optimize the model performance, but rather learn about the predictive power of each algorithm. In addition, for every embedding algorithm, we run that algorithm 10 times with the same parameters. This is done to build a distribution over embeddings, since random-walk based algorithms have stochastic nature to them. Furthermore, we train our classifier (built using Neural Network model) 10 times to capture appropriate statistic over measured metrics. We randomly initialize our neural-network model and use drop-out and $L_1$ and $L_2$ regularization. All models are train for 50 epochs using standard learning rate.

As highlighted, using carefully designed node features (as noted in the previous section), one could achieve accuracy of $66\%$ in detecting bot vs. non-bot accounts. As we highlighted in Fig. 23, features such as *pagerank* and *coreness* show predictive power as compared with other node features. One could use this information to design similar features related to node centrality and coreness to capture additional predictive power, however this process is time consuming and could miss important features. As we have suggested, a different approach is to leverage unsupervised machine learning techniques to capture various properties on nodes, without time consuming featuring engineering. As we show in this section, one could utilize node and structural embedding to learn different types of representations of node, and combine them to gain even greater predictive power.

**Table 5:** Bot classification performance summary for the **Italian-Election** dataset

Feature-Set	Accuracy	Precision	Recall	f1-Score	MCC
Node Features	0.66	0.65	0.66	0.66	0.31
All Embeddings	0.69	0.70	0.64	0.67	0.38
Node2Vec	0.61	0.62	0.57	0.59	0.22
DeepWalk	0.62	0.62	0.59	0.60	0.23
LSME	0.64	0.64	0.65	0.64	0.29
Struc2Vec	0.63	0.66	0.55	0.60	0.27
GraphWave	0.64	0.65	0.61	0.63	0.29
Role2Vec	0.67	0.68	0.61	0.65	0.34

We see in Fig. 21 that models trained exclusively on features extracted using embeddings perform inline with a model trained using engineered node features. Node embeddings, such as Node2Vec and DeepWalk, could learn information about the node’s local community and proximity, while structural embeddings could learn information about the local structure of the network around each node. As we can see, a combination of these features could capture a broader representation of nodes, and in fact perform as well, or even better than a model trained on node features. In this case, *All Embedding* is a model trained on a feature set built by combining all the embeddings together. We note that to make our comparison fair, we apply Principal Component Analysis (PCA) to this combined feature set to reduce the dimensionality to 64 (same as embedding dimension of the embedding algorithms). We also highlight the fact that models trained using *Role2Vec* embedding outperforms all other embedding, indicating that for the **Italian Election** dataset, the structural property of the nodes is a better indicator if an account is bot vs. non-bot. Other metrics in presented in Table 5 tell similar stories, where models trained on all embedding perform best in our study. Lastly, we point out that the comparison in Fig. 21 is designed to analyze predictive performance of various embedding algorithms against one another and models train using node features exclusively. The presented results highlight the fact that unsupervised embedding techniques (such as the ones studied here) can be used to extract features from the underlying social network that have predictive power when it comes to tasks such as bot detection. A comparison between embeddings and other types of features is shown in Table 5.

**Fig. 22:**
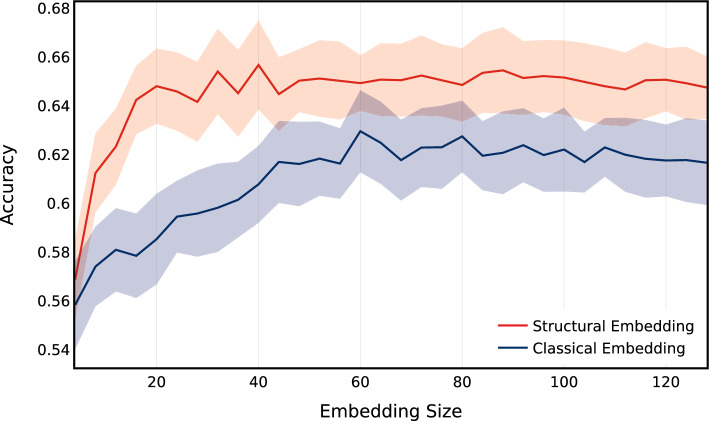
Accuracy as a function of embedding size for structural and classical embeddings. Simulations performed using the **Italian Election** dataset. The solid line correspond to the mean accuracy for 50 runs, and the band correspond to the standard deviation of the results

To understand why features generated from embedding algorithms have predictive power for bot classification, one needs to focus on the behaviour of bots in a social network. Similar to real users, bots interact with the social network by following, tweeting and engaging with users. Most of this activity requires engagement from real users in addition to the actions that bot accounts take. One could measure this by studying the structure of the local network surrounding each user. For example who each user is connected to, and who their friends are connected to and so on. Although it may be trivial for bot accounts to generate human realistic content and profile, given the current advances in machine learning, it is much more difficult to encourage other users to interact with the content in an organic way. This manifests itself in how the local structure of the social network around each is formed. We use this fact as motivation to capture predictive features which could help us identify bot accounts. In Fig. 21 we show that these structural properties have predictive power by both creating structural features manually using node structural features (Node Feature) and by capturing them using structural embedding algorithms (LSME, Struc2Vec, GraphWave, and Role2Vec). This idea is further emphasized by the fact that classical embedding algorithms, such as Node2Vec and DeepWalk, do not perform as well, since they capture information about the proximity rather than structural. In Fig. 22 we present a comparison of classical vs structural embeddings performance as a function of embedding dimension. Similar to the **TwiBot-20** case structural embeddings are consistently better than classical ones and increasing embedding dimension above 35 does not improve predictive performance.

**Fig. 23:**
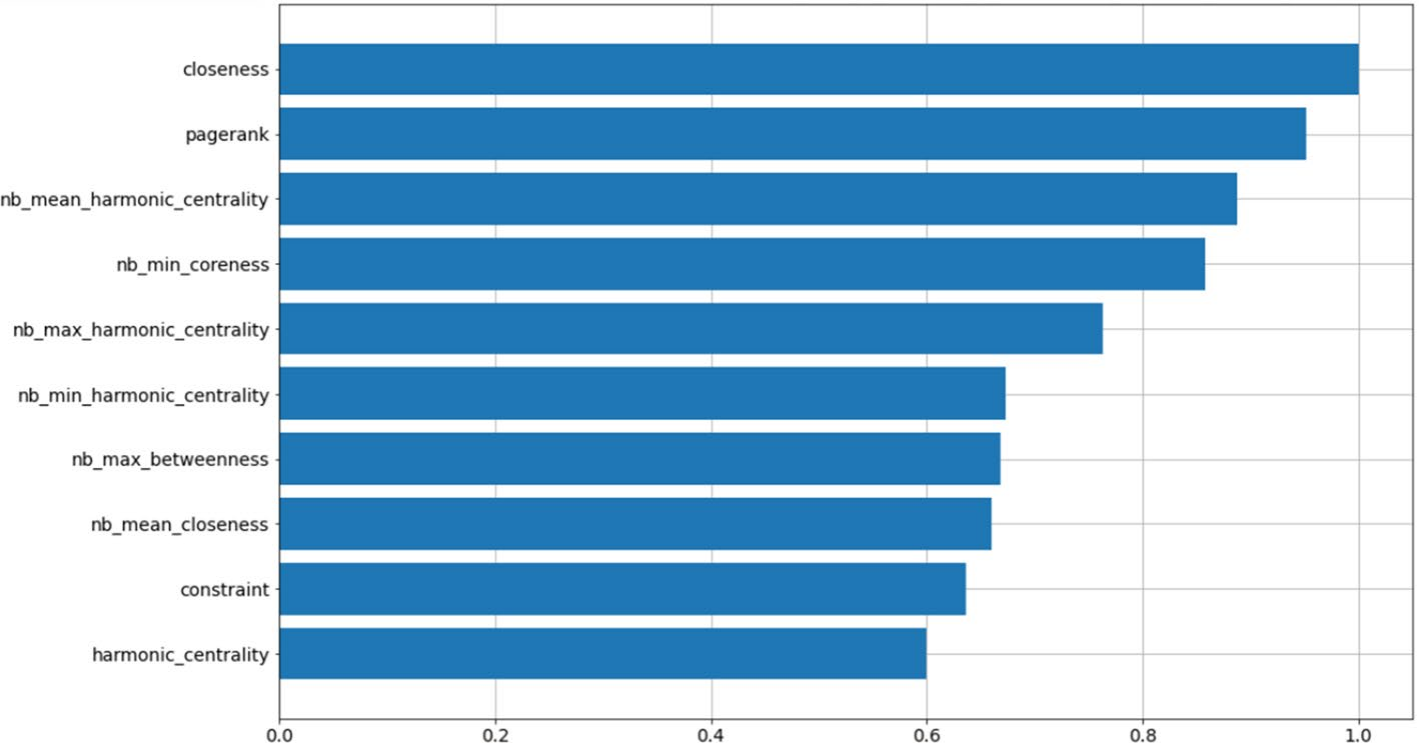
Variable importance for the H2O AutoML leader model built on the basis of node features (exclusively) (**Italian-Election** dataset)

Lastly, we perform feature importance analysis on the **GF** feature-set. The results are presented in Fig. 23. Similar to the **TwiBot-20** dataset, *pagerank* appear in the top two most predictive features. Given the difference in the topology of the two graphs, **TwiBot-20** and **Italian Election**, it is interesting to observe *pagerank* appearing as one of the most important features. However, unlike **TwiBot-20**, we observe a number of important features derived from statistics of node-features from neighbouring nodes of each vertex. This indicates that identifying bots using node-features requires more than local properties of nodes. This further supports the use of embeddings, since many embedding algorithms can learn global properties of nodes.

We conclude this section by emphasizing that the performance of each embedding on this dataset (**Italian-Election**) is not an indication that they perform in similar range if applied to other datasets. In general, embeddings are very application specific. This can also be said about hand-designed node features. Given the diversity amongst datasets, one should aim to use techniques that learn a wide range of representation of nodes in an unsupervised way. For this reason, an embedding technique (or a combination of them) could be power tool that could generalize well across various applications.

## Predictive stability

One of the main challenges with building scalable and stable machine learning systems is understanding the impact of data noise on the outcome of the model. One would expect that as the level of noise (feature variability due to randomness) increases, the performance of the model would decline. To combat model degradation due to noise, one could either design or search for models that are resilient to randomness, or engineer features with better signal to noise ratio. In this section, we aim in investigate the impact of synthetically added noise on the performance of the bot detection classifier. The idea is to learn whether features extracted via embedding algorithms could perform as well as engineered features when subject to increase in randomness in the system. To study the stability of the features, we use the **Italian Election** dataset and incrementally increase the amount of noise in the system by randomly adding and removing edges between the nodes. We measure the noise on a scale that ranges from $\alpha =0$ to $\alpha =100$. The process of introducing noise in the system is as follows. For every edge in the original network (for example $e_{ab}$), we compute a random number *p* between 0 and 100. If $p\le \alpha$ we remove the edge $e_{ab}$, and add another edge between one of the original nodes (*a* or *b*) to another random node in the graph (e.g. $e_{ab} \rightarrow e_{ac}$). In this process, $\alpha =0$ corresponds to the original graph, and $\alpha =100$ corresponds to a graph where every edge is replaced with a random edge. This process mimics a synthetic addition and removal of follower-following relationships. For every iteration (level of added noise), we embed every node in the graph using the selected algorithms and use the resulting features to build a model for identifying bots. Here, we use the model ROC-AUC to measure the impact of increase in randomness on the system. It is important to note that for systems with randomness of $\alpha =100$ there are still structures that could have predictive power when it comes to detecting bots, meaning that $\alpha =100$ does not correspond to a random graph. Users (nodes) with large number of friends (high degree) would still have many friends, but their local network structure would have been randomized.

**Fig. 24:**
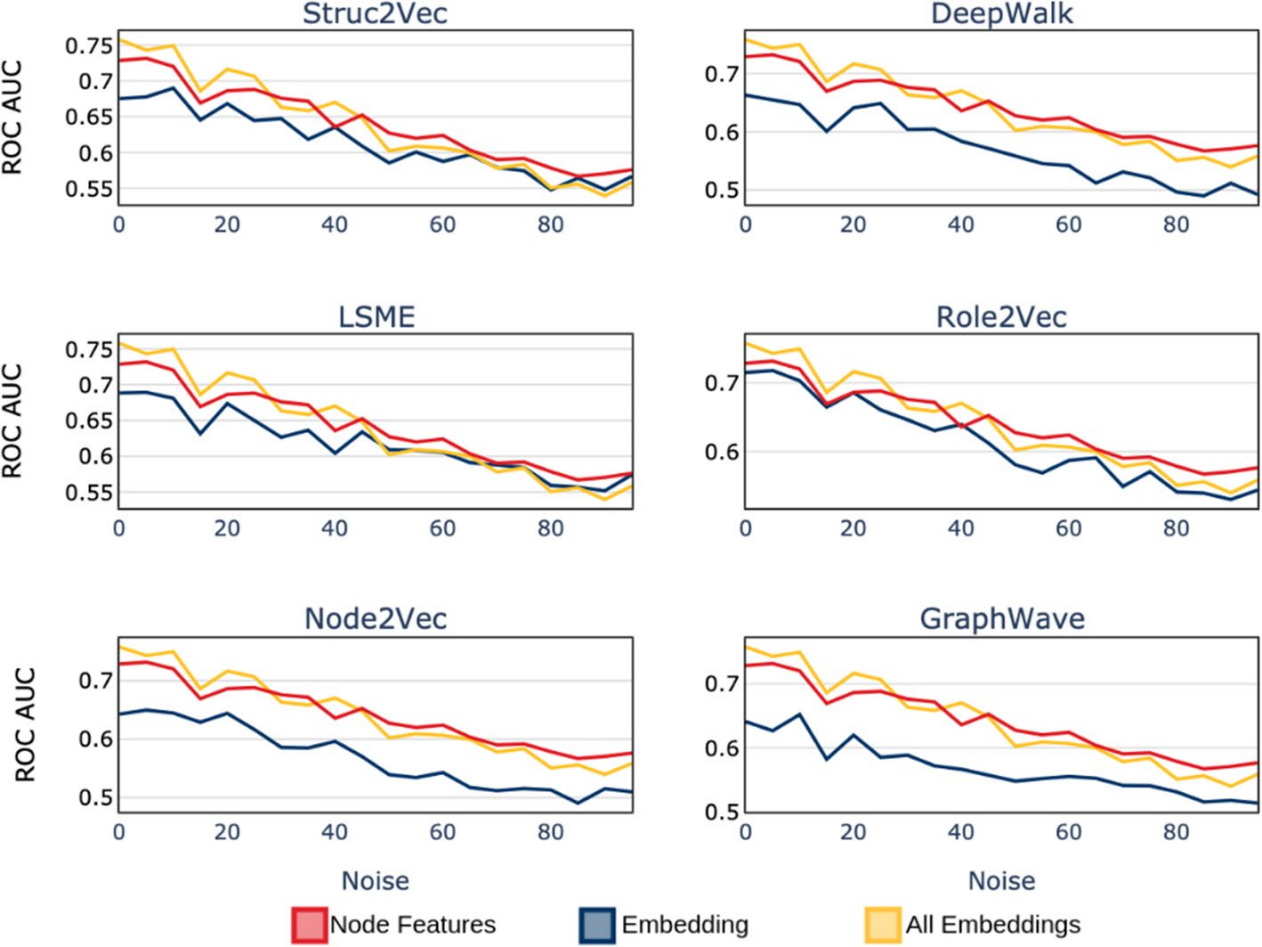
Stability of ROC-AUC score: ROC-AUC of node features (red), ROC-AUC of specific embedding (blue), and ROC-AUC of all embeddings combined (yellow)

The results for our simulations are presented in Fig. 24. As expected, increase in the level of noise in the system results in a decline in the quality of features and thus a poorer performing model. For every sub-plot, the ROC-AUC for models built using node-features in plotted in red, those built using selected embedding algorithm plotted in blue, and the combined embedding feature set in yellow. Based on our simulations, features built using all embedding algorithms decline in performance at the same rate as models built using node-features. This highlights the fact that node representations leaned by embeddings are generally as resilient to noise, as hand-crafted features. The utility of using unsupervised embedding algorithms for learning node-representations is shown to be resilient to added noise in the network. This is important since the underlying network (Twitter network in this case) is dynamic and changes overtime. Nodes that may be connected at a given snapshot in time may not be connected at later dates. Therefore, the local and global structure of the network is constantly changing and therefore models and features need to be flexible to adjust to the dynamic of the system.

## Conclusion

In this work, we examined four distinct feature-sets extracted from the Twitter social network for identifying bot accounts. We divided the features into those captured directly from the Twitter network, NLP and user-profile data (**NLP** and **P**), and those derived from the underlying network structure, node-features (**GF**) and embeddings (**EMB**). The main aim of the analysis was to understand the performance of embeddings in this task. As an initial data analysis task we verified, following the earlier literature, that NLP and user profile features have strong predictive power. However, we argue that they may suffer from the lack of generalizability. For example, language models trained for identifying bot accounts in an English speaking region can not be used to other regions. Similarly, clever bot accounts can modify their user profiles to appear more natural (non-bot like), and avoid being detected as bots. A much more difficult task however is altering the topology of the social network surrounding an account to appear as if it was created organically. Building on this intuition, we showed that the features extracted by mining graph structures indeed holds predictive power in helping us identify bots.

We analyzed features extracted from two Twitter datasets, one (**Twibot-20**) built using the follower as well as following relationships between users, and the other (**Italian Election**) constructed based on the interactions between users. We saw that in both networks, features mined from the underlying graph, either through node-feature engineering or learned in an unsupervised way via embedding algorithms, have predictive power. This positively verifies our first hypothesis that bot behaviour in a social network is distinguishable from non-bot users in both how they (bots) build relationships with other users and how they interact with them.

We compared usefulness for bot prediction of two types of embeddings: classical and structural. Our findings show that structural embeddings consistently perform better than classical ones. Additionally we found that for bot detection task using very-highly dimensional embeddings is not needed. For both analyzed data sets beyond 35 dimensions the predictive power of embeddings stopped increasing. As an additional finding we showed that one can combine features learned from various node and structural embeddings to a hybrid feature set. Such an approach improves prediction quality, which show that different embeddings capture different network characteristics and that contain complementary information for bot detection.

Finally, we showed that using embeddings is as resistant to noise in data as using hand-crafted node features, which shows that this approach can be useful for networks that are dynamic and evolving over time. The **TwiBot-20** and **Italian-Election** graphs are significantly different in their construction and interpretation, although in both of them nodes represent users. However, for both of them we found embeddings to be a useful tool for bot detection. Therefore an interesting future research question would be to study the impact of combining features gathered various network definitions, for example one built on follower/following and another on user-user interaction, for identifying bots.

## Data Availability

The two datasets that are analyzed (**TwiBot-20** and **Italian Election**) are publicly available—see "Datasets" section for more details. Jupyter notebook containing all experiments performed in the paper is available from the corresponding author on request.

## Appendix

See Tables 6 and 7

**Table 6:** Statistics for the **Twibot-20** dataset node features

	KS p-value	Mean		STD		Median	
		Not-Bot	Bot	Not-Bot	Bot	Not-Bot	Bot
$degree\_centrality$	4.943e–44	9.343e–05	1.038e–04	3.744e–05	1.823e–05	1.096e–04	1.096e–04
$eigen\_centrality$	6.661e–15	2.197e–03	1.609e–03	1.081e–02	5.991e–03	3.455e–04	2.455e–04
*authority*	6.661e–15	3.907e–03	2.857e–03	1.971e–02	1.092e–02	5.899e–04	4.249e–04
*closeness*	6.921e–26	1.621e–01	1.557e–01	1.478e–01	1.376e–01	1.357e–01	1.326e–01
*betweenness*	4.359e–13	1.159e+07	8.453e+06	3.630e+07	1.332e+07	4.197e+06	4.145e+06
*constraint*	3.140e–44	1.106e–01	6.239e–02	1.770e–01	8.422e–02	4.762e–02	4.762e–02
*coreness*	6.661e–15	2.133e+00	2.039e+00	7.300e–01	6.973e–01	2.000e+00	2.000e+00
*eccentricity*	6.022e–19	1.239e+01	1.260e+01	2.193e+00	2.054e+00	1.300e+01	1.300e+01
$harmonic\_centrality$	1.880e–22	1.301e–01	1.285e–01	2.682e–02	2.413e–02	1.330e–01	1.304e–01
*pagerank*	1.030e–45	3.936e–05	4.427e–05	1.596e–05	8.721e–06	4.620e–05	4.667e–05
$nb\_mean\_degree\_centrality$	8.629e–66	3.435e–05	2.045e–05	3.757e–05	2.390e–05	1.424e–05	1.143e–05
$nb\_min\_degree\_centrality$	1.870e–57	2.058e–05	7.918e–06	3.346e–05	1.576e–05	5.220e–06	5.220e–06
$nb\_max\_degree\_centrality$	7.669e–05	1.115e–04	1.147e–04	6.168e–05	6.732e–05	1.096e–04	1.096e–04
$nb\_std\_degree\_centrality$	1.786e–34	2.482e–05	2.699e–05	1.720e–05	1.812e–05	2.278e–05	2.278e–05
$nb\_mean\_eigen\_centrality$	7.283e–46	1.657e–03	1.111e–03	4.441e–03	4.378e–03	2.899e–04	1.534e–04
$nb\_min\_eigen\_centrality$	1.809e–49	3.513e–04	1.642e–04	1.658e–03	7.329e–04	4.484e–05	2.256e–05
$nb\_max\_eigen\_centrality$	1.711e–12	1.407e–02	1.302e–02	4.770e–02	5.357e–02	2.529e–03	1.835e–03
$nb\_std\_eigen\_centrality$	3.918e–26	3.551e–03	3.109e–03	1.086e–02	1.278e–02	6.618e–04	4.372e–04
$nb\_mean\_authority$	4.629e–45	2.948e–03	1.975e–03	8.057e–03	7.930e–03	5.009e–04	2.624e–04
$nb\_min\_authority$	4.315e–48	6.211e–04	2.904e–04	3.000e–03	1.297e–03	7.740e–05	3.840e–05
$nb\_max\_authority$	2.780e–12	2.522e–02	2.324e–02	8.752e–02	9.805e–02	4.401e–03	3.122e–03
$nb\_std\_authority$	4.177e–25	6.357e–03	5.545e–03	1.988e–02	2.334e–02	1.147e–03	7.546e–04
$nb\_mean\_closeness$	2.358e–64	1.383e–01	1.316e–01	6.769e–02	6.307e–02	1.252e–01	1.203e–01
$nb\_min\_closeness$	2.352e–64	1.342e–01	1.283e–01	6.798e–02	6.335e–02	1.217e–01	1.174e–01
$nb\_max\_closeness$	1.973e–19	1.627e–01	1.589e–01	6.488e–02	5.961e–02	1.512e–01	1.488e–01
$nb\_std\_closeness$	2.381e–09	8.135e–03	8.000e–03	3.175e–03	2.270e–03	8.072e–03	7.936e–03
$nb\_mean\_betweenness$	9.559e–45	8.612e+06	6.439e+06	1.395e+07	1.386e+07	3.244e+06	2.797e+06
$nb\_min\_betweenness$	3.294e–57	1.618e+06	2.413e+05	6.376e+06	2.046e+06	0.000e+00	0.000e+00
$nb\_max\_betweenness$	1.554e–15	7.376e+07	8.260e+07	1.399e+08	1.603e+08	4.678e+07	4.851e+07
$nb\_std\_betweenness$	2.458e–13	1.866e+07	1.974e+07	3.275e+07	3.919e+07	1.136e+07	1.120e+07
$nb\_mean\_constraint$	1.489e–56	6.876e–01	8.155e–01	3.463e–01	2.185e–01	8.615e–01	8.854e–01
$nb\_min\_constraint$	5.360e–09	9.423e–02	8.746e–02	1.783e–01	1.661e–01	4.762e–02	4.762e–02
$nb\_max\_constraint$	1.870e–57	8.220e–01	9.699e–01	3.657e–01	1.625e–01	1.000e+00	1.000e+00
$nb\_std\_constraint$	9.267e–57	2.307e–01	2.669e–01	1.295e–01	9.025e–02	2.562e–01	2.728e–01
$nb\_mean\_coreness$	5.296e–58	1.558e+00	1.325e+00	6.183e–01	3.437e–01	1.286e+00	1.200e+00
$nb\_min\_coreness$	1.810e–52	1.242e+00	1.035e+00	5.561e–01	2.246e–01	1.000e+00	1.000e+00
$nb\_max\_coreness$	3.123e–01	2.755e+00	2.745e+00	5.408e–01	5.244e–01	3.000e+00	3.000e+00
$nb\_std\_coreness$	5.627e–08	5.106e–01	5.357e–01	2.499e–01	2.175e–01	5.118e–01	5.145e–01
$nb\_mean\_eccentricity$	6.843e–59	1.302e+01	1.339e+01	2.180e+00	2.035e+00	1.359e+01	1.381e+01
$nb\_min\_eccentricity$	4.205e–11	1.161e+01	1.179e+01	1.876e+00	1.757e+00	1.200e+01	1.200e+01
$nb\_max\_eccentricity$	1.056e–52	1.325e+01	1.357e+01	2.195e+00	2.056e+00	1.400e+01	1.400e+01
$nb\_std\_eccentricity$	5.160e–10	4.977e–01	4.892e–01	1.921e–01	1.574e–01	4.781e–01	4.781e–01
$nb\_mean\_harmonic\_centrality$	2.640e–60	1.206e–01	1.164e–01	2.587e–02	2.202e–02	1.221e–01	1.177e–01
$nb\_min\_harmonic\_centrality$	9.593e–61	1.164e–01	1.130e–01	2.419e–02	2.081e–02	1.184e–01	1.147e–01
$nb\_max\_harmonic\_centrality$	1.554e–15	1.459e–01	1.449e–01	2.974e–02	2.742e–02	1.497e–01	1.474e–01
$nb\_std\_harmonic\_centrality$	7.633e–09	8.423e–03	8.349e–03	2.776e–03	2.289e–03	8.416e–03	8.275e–03
$nb\_mean\_pagerank$	5.759e–68	1.374e–05	8.420e–06	1.456e–05	9.474e–06	5.861e–06	4.926e–06
$nb\_min\_pagerank$	1.009e–67	8.174e–06	3.664e–06	1.225e–05	5.819e–06	2.744e–06	2.708e–06
$nb\_max\_pagerank$	7.704e–44	4.215e–05	4.254e–05	2.019e–05	2.163e–05	4.192e–05	4.073e–05
$nb\_std\_pagerank$	9.750e–24	9.424e–06	9.844e–06	5.811e–06	6.044e–06	8.424e–06	8.401e–06
$in\_degree\_centrality$	3.201e–36	5.101e–05	5.159e–05	2.302e–05	1.122e–05	5.220e–05	5.220e–05
$out\_degree\_centrality$	3.822e–167	4.280e–05	5.283e–05	2.122e–05	1.013e–05	5.220e–05	5.220e–05
$in\_degree$	3.201e–36	9.771e+00	9.883e+00	4.409e+00	2.149e+00	1.000e+01	1.000e+01
$out\_degree$	3.822e–167	8.198e+00	1.012e+01	4.065e+00	1.940e+00	1.000e+01	1.000e+01

**Table 7:** Statistics for the **Italian Election** dataset node features

	KS p–value	Mean		STD		Median	
		Not-Bot	Bot	Not-Bot	Bot	Not-Bot	Bot
$degree\_centrality$	1.332e–15	2.600e–04	2.874e–04	2.572e–03	1.489e–03	8.063e–05	8.063e–05
*authority*	1.000e+00	1.298e–04	1.024e–04	1.134e–02	8.611e–05	2.221e–17	1.748e–04
*closeness*	1.000e+00	2.993e–01	4.539e–01	2.292e–01	2.093e–01	2.172e–01	5.001e–01
*betweenness*	1.332e–15	7.929e+03	1.356e+04	5.707e+04	1.347e+05	0.000e+00	0.000e+00
$hub\_score$	1.000e+00	1.298e–04	1.024e–04	1.134e–02	8.611e–05	2.221e–17	1.748e–04
*constraint*	1.332e–15	7.483e–01	8.606e–01	3.367e–01	2.930e–01	1.000e+00	1.000e+00
*coreness*	1.332e–15	1.986e+00	1.474e+00	2.042e+00	1.561e+00	1.000e+00	1.000e+00
*eccentricity*	1.000e+00	8.513e+00	4.277e+00	3.151e+00	3.701e+00	1.000e+01	2.000e+00
$harmonic\_centrality$	1.000e+00	1.159e–01	1.049e–01	5.474e–02	4.228e–02	1.363e–01	1.111e–01
*pagerank*	1.000e+00	7.513e–05	8.992e–05	1.160e–03	3.302e–04	4.123e–05	4.359e–05
$nb\_mean\_degree\_centrality$	1.000e+00	9.788e–03	1.314e–01	2.077e–02	1.080e–01	3.697e–03	2.221e–01
$nb\_min\_degree\_centrality$	1.000e+00	7.782e–03	1.311e–01	2.087e–02	1.084e–01	1.129e–03	2.221e–01
$nb\_max\_degree\_centrality$	1.000e+00	1.307e–02	1.322e–01	2.198e–02	1.071e–01	5.563e–03	2.221e–01
$nb\_std\_degree\_centrality$	1.332e–15	6.683e–03	2.365e–03	6.537e–03	3.973e–03	4.903e–03	7.982e–04
$nb\_mean\_authority$	1.000e+00	7.069e–03	5.861e–01	8.378e–02	4.926e–01	7.639e–16	1.000e+00
$nb\_min\_authority$	1.000e+00	7.069e–03	5.861e–01	8.378e–02	4.926e–01	2.132e–16	1.000e+00
$nb\_max\_authority$	1.000e+00	7.069e–03	5.861e–01	8.378e–02	4.926e–01	1.124e–15	1.000e+00
$nb\_std\_authority$	1.332e–15	1.321e–15	4.721e–16	1.271e–15	7.874e–16	9.460e–16	1.582e–16
$nb\_mean\_closeness$	1.000e+00	3.573e–01	7.583e–01	2.622e–01	3.518e–01	2.594e–01	1.000e+00
$nb\_min\_closeness$	1.000e+00	3.464e–01	7.525e–01	2.656e–01	3.595e–01	2.471e–01	1.000e+00
$nb\_max\_closeness$	1.000e+00	3.689e–01	7.660e–01	2.605e–01	3.422e–01	2.742e–01	1.000e+00
$nb\_std\_closeness$	1.332e–15	2.897e–02	3.192e–02	2.835e–02	3.601e–02	2.395e–02	2.580e–02
$nb\_mean\_betweenness$	1.000e+00	6.903e+05	2.331e+06	9.976e+05	1.785e+06	2.028e+05	3.794e+06
$nb\_min\_betweenness$	1.000e+00	5.158e+05	2.298e+06	9.820e+05	1.818e+06	3.925e+04	3.794e+06
$nb\_max\_betweenness$	1.000e+00	1.025e+06	2.418e+06	1.307e+06	1.735e+06	3.349e+05	3.794e+06
$nb\_std\_betweenness$	1.332e–15	6.359e+05	2.543e+05	6.725e+05	4.793e+05	4.200e+05	7.926e+04
$nb\_mean\_hub\_score$	1.000e+00	7.069e–03	5.861e–01	8.378e–02	4.926e–01	7.639e–16	1.000e+00
$nb\_min\_hub\_score$	1.000e+00	7.069e–03	5.861e–01	8.378e–02	4.926e–01	2.132e–16	1.000e+00
$nb\_max\_hub\_score$	1.000e+00	7.069e–03	5.861e–01	8.378e–02	4.926e–01	1.124e–15	1.000e+00
$nb\_std\_hub\_score$	1.332e–15	1.321e–15	4.721e–16	1.271e–15	7.874e–16	9.460e–16	1.582e–16
$nb\_mean\_constraint$	1.000e+00	1.923e–01	1.673e–01	2.834e–01	3.030e–01	5.828e–02	3.630e–04
$nb\_min\_constraint$	1.000e+00	1.489e–01	1.208e–01	2.757e–01	2.743e–01	2.362e–02	3.630e–04
$nb\_max\_constraint$	1.000e+00	2.660e–01	2.239e–01	3.516e–01	3.794e–01	8.484e–02	3.630e–04
$nb\_std\_constraint$	1.332e–15	1.427e–01	2.290e–01	1.698e–01	1.794e–01	6.639e–02	2.427e–01
$nb\_mean\_coreness$	1.000e+00	6.150e+00	2.254e+00	3.997e+00	2.689e+00	6.688e+00	1.000e+00
$nb\_min\_coreness$	1.000e+00	5.190e+00	1.839e+00	4.016e+00	2.336e+00	4.000e+00	1.000e+00
$nb\_max\_coreness$	1.000e+00	6.918e+00	2.752e+00	4.465e+00	3.535e+00	8.000e+00	1.000e+00
$nb\_std\_coreness$	1.332e–15	2.131e+00	1.956e+00	1.869e+00	1.836e+00	2.041e+00	1.708e+00
$nb\_mean\_eccentricity$	1.000e+00	7.858e+00	3.543e+00	3.018e+00	3.868e+00	9.000e+00	1.000e+00
$nb\_min\_eccentricity$	1.000e+00	7.641e+00	3.397e+00	2.948e+00	3.658e+00	9.000e+00	1.000e+00
$nb\_max\_eccentricity$	1.000e+00	8.065e+00	3.652e+00	3.124e+00	4.037e+00	9.000e+00	1.000e+00
$nb\_std\_eccentricity$	1.332e–15	5.647e–01	5.983e–01	3.995e–01	4.102e–01	5.774e–01	5.964e–01
$nb\_mean\_harmonic\_centrality$	1.000e+00	1.425e–01	1.754e–01	6.839e–02	7.325e–02	1.677e–01	2.221e–01
$nb\_min\_harmonic\_centrality$	1.000e+00	1.348e–01	1.720e–01	6.665e–02	7.481e–02	1.523e–01	2.221e–01
$nb\_max\_harmonic\_centrality$	1.000e+00	1.505e–01	1.802e–01	7.232e–02	7.251e–02	1.767e–01	2.221e–01
$nb\_std\_harmonic\_centrality$	1.332e–15	1.960e–02	1.792e–02	1.241e–02	1.241e–02	1.909e–02	1.839e–02
$nb\_mean\_pagerank$	1.000e+00	2.587e–03	6.013e–02	8.740e–03	4.995e–02	8.351e–04	1.021e–01
$nb\_min\_pagerank$	1.000e+00	2.156e–03	6.006e–02	8.771e–03	5.003e–02	2.732e–04	1.021e–01
$nb\_max\_pagerank$	1.000e+00	3.318e–03	6.030e–02	8.864e–03	4.975e–02	1.169e–03	1.021e–01
$nb\_std\_pagerank$	1.332e–15	1.482e–03	5.014e–04	1.556e–03	8.927e–04	1.017e–03	1.503e–04

## Footnotes

1 Redundant feature—it may be computed from the other features but it is explicitly included in the model.
